# Hybrid Composite of Sn(IV)-Porphyrin and Mesoporous Structure for Enhanced Visible Light Photocatalytic Degradation of Organic Dyes

**DOI:** 10.3390/molecules28041886

**Published:** 2023-02-16

**Authors:** Nirmal Kumar Shee, Beom-Hyeok Park, Hee-Joon Kim

**Affiliations:** Department of Chemistry and Bioscience, Kumoh National Institute of Technology, Gumi 39177, Republic of Korea

**Keywords:** hybrid composite, Sn(IV)-porphyrin, mesoporous structure, photocatalytic degradation, water remediation

## Abstract

Two hybrid composites (**SnP@MCM−41** and **SnP@SiO_2_**) were fabricated by chemical adsorption of (*trans*-dihydroxo)(5,10,15,20-tetraphenylporphyrinato)tin(IV) (**SnP**) on mesoporous structured Mobil Composition of Matter No. 41 (MCM−41) and SiO_2_ nanoparticles. These materials were characterized by Fourier-transform infrared spectroscopy, ultraviolet–visible spectroscopy, fluorescence spectroscopy, transmission electron microscopy, and field-emission scanning electron microscopy techniques. The incorporation of **SnP** into MCM−41 and SiO_2_ supports efficient photocatalytic degradation of the anionic erioglaucine, cationic rhodamine B, and neutral *m*-cresol purple dyes under visible light irradiation in an aqueous solution. The performances of degradation of these dyes by these photocatalysts under visible light irradiation varied from 87 to 95%. The pseudo-first-order degradation rate constant of organic dyes for **SnP@MCM−41** was higher than those of **SnP@SiO_2_** and **SnP**. These visible light photocatalysts showed remarkable stability and reliable reusability.

## 1. Introduction

The global environment suffers from water pollution due to increased industrial and agricultural activities, which causes serious problems for aquatic life [1,2]. Every year, large amounts of hazardous substances such as dyes, pesticides, pigments, herbicides, phenols, biphenyls, and nitro and amino compounds are discharged into water bodies, impairing the drinking quality of water. In this regard, scientific efforts to remove these toxic substances from wastewater are increasing [3,4]. Numerous physicochemical methods including adsorption [5], filtration [6], precipitation [7], and advanced oxidation processes (AOPs) [8,9] have been employed to remove these pollutants. AOP is the most suitable technology because it is simple, has a low cost and high degradation rate, and degrades the entire pollutant to nontoxic H_2_O and CO_2_ without generation of other secondary pollutants. Appropriate photocatalysts in the AOPs absorb visible light from the sun and generate reactive oxygen species in situ, which promote the degradation of chemicals in water. A large number of photocatalysts have been used to analyze the degradation of organic pollutants in water. Porphyrinoids (free-base porphyrins and metalloporphyrins) largely absorb visible light and can be used as visible-light-activated photocatalysts [10,11]. However, the use of porphyrinoids in homogeneous catalysis is limited by their aggregation in solution, small surface area, poor reusability, and difficulty in postseparation of the catalyst from the reaction mixture [12]. In addition, it is necessary to develop new photocatalysts to overcome main challenges such as the reduced bandgap, increased solar absorption, fast electron–hole recombination, and smooth recovery from the reaction medium. To this end, porphyrin-based hybrid composite materials have been promising photocatalysts for the degradation of organic pollutants in water and have attracted considerable attention in the applied chemistry community over the past decades [13,14,15,16,17]. In this method, porphyrinoids are combined with one or more components, leading to the formation of nonaggregated nanomaterials with controlled surface morphologies. These materials exhibit remarkable features compared to starting compounds, including a large surface area, high physiochemical stability, and morphology-dependent physical properties.

**Scheme 1:**
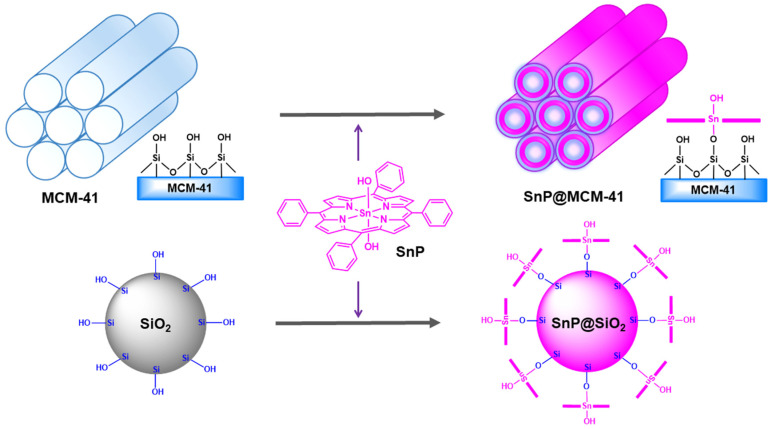
Fabrication of the hybrid composite **SnP@MCM−41** and **SnP@SiO_2_**.

In this context, we have been interested in hybrid nanocomposite materials comprising metalloporphyrins and mesoporous supports. In this study, a mesoporous Mobil Composition of Matter No. 41 (MCM−41), which consists of a hexagonally well-ordered structure with large pore size and surface area, was used. The numerous silanol groups on the cavity surface attract other functional groups for chemical or physical adsorption [18,19,20]. Commercial SiO_2_ nanoparticles were used for comparative studies. We selected Sn(IV)-porphyrins as a source of porphynoid compounds. Sn(IV)-porphyrin was selected over others owing to its unique optical properties and peculiar octahedral geometry. Owing to the oxophilic nature of the Sn(IV) center, Sn(IV)-porphyrin can readily form stable six-coordinated complexes with two oxyanions of alkoxides or carboxylates at the *trans* position. This advantageous tuning capability of Sn(IV)-porphyrins enables creation of coordination complexes [21,22,23,24,25], multiporphyrin arrays [26,27,28], and nanostructures [29,30,31], which exhibit characteristic functions. In particular, supramolecular nanostructures based on Sn(IV)-porphyrins have been intensively studied for the application of photocatalysis of AOPs for water remediation [32,33,34,35,36,37,38,39,40]. Therefore, the combination of Sn(IV)-porphyrins with the mesoporous MCM−41 not only enables morphology fabrication of the surface but also enhances the photocatalytic degradation efficiencies of organic pollutants by manifold compared to the parent Sn(IV)-porphyrins (Scheme 1). As model organic pollutants, anionic erioglaucine (EG) [41], cationic rhodamine B (RhB) [42], and neutral *m*-cresol purple (MCP) [43] dyes were chosen to investigate photocatalytic degradation. These pollutants are potentially carcinogenic even at very low concentrations, non-biodegradable, and long-lasting in aqueous solutions. These water-soluble dyes have been widely used in the textile, paper-printing, and leather industries and are discharged in significant amounts to the environment. This results in undesirable water pollution, and thus it is required to remove these dyes from wastewater.

## 2. Results and Discussion

### 2.1. Fabrication and Characterization

Hybrid composites (**SnP@MCM−41** or **SnP@SiO_2_**) composed of Sn(IV)-porphyrin and support materials were fabricated through the reaction of **SnP** with silanol groups of mesoporous materials. Condensation of the axial hydroxo ligand of Sn(IV)-porphyrin with a silanol group of the silica surface forms Sn−O−Si chemical bonds in the fabrication of the hybrid composites, as shown in Scheme 1. In a typical procedure, **SnP** was reacted with MCM-41 or SiO_2_ in dichloromethane at room temperature for 24 h, and then the solid was filtered to obtain a hybrid composite of **SnP@MCM-41** or **SnP@SiO_2_**. The amount of adsorbed Sn(IV)-porphyrin (**SnP**) was estimated based on the tin content of the hybrid composite, **SnP@MCM−41** or **SnP@SiO_2_**, measured by ICP analysis. The chemically adsorbed amounts of **SnP** in **SnP@MCM−41** and **SnP@SiO_2_** were estimated to be 0.094 and 0.091 mmol/g, respectively.

The surface modification was characterized by zeta potentials determined by the degree of electrostatic repulsion of the surface. As shown in Table S1, the average zeta potentials of SiO_2_ and MCM-41, and **SnP** are −23.80 and −30.66 mV, respectively. On the other hand, the average zeta potentials of **SnP@SiO_2_** and **SnP@MCM−41** are −17.56 and −27.53 mV, respectively. The positively increased zeta potentials for **SnP@SiO_2_** and **SnP@MCM−41** compared to those for bare SiO_2_ and MCM−41 show the strong binding of **SnP** with SiO_2_ and MCM−41, which indicates the formation of desired hybrid composites.

**Figure 1:**
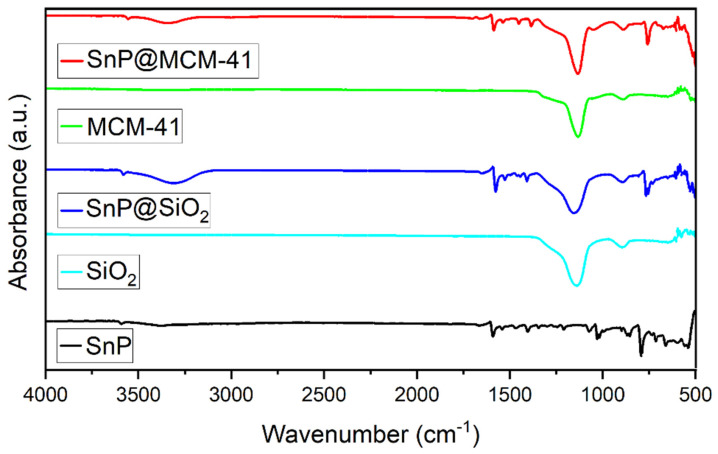
FTIR spectra of **SnP**, SiO_2_, **SnP@SiO_2_**, MCM−41, and **SnP@MCM−41**.

FTIR spectra of **SnP@MCM−41** and **SnP@SiO_2_** were compared to those of **SnP**, SiO_2_, and MCM−41, as shown in Figure 1. In the FTIR spectrum of **SnP**, the absorption peaks at 1021 and 796 cm^−1^ belong to the bending vibration of N−H and out-of-plane bending vibration of C−H in the benzene ring, respectively. The peaks at 3381, 1590, and 1409 cm^−1^ are attributed to the stretching vibrations of N−H, C=C, and C−N in the pyrrole ring, respectively. The peaks at 3600 cm^−1^ are assigned to the stretching vibrations of the OH signal of the axial hydroxo ligand present in the Sn(IV)-porphyrin. The absorption peaks at 1052 and 805 cm^−1^ are characteristic peaks of Si−O−Si stretching, while the peaks at 470 and 950 cm^−1^ are assigned to the bending of Si−O−Si and Si−OH for MCM−41, respectively. On the other hand, the peaks at 1063 and 803 cm^−1^ are characteristic peaks of Si−O−Si stretching, while those at 472 and 949 cm^−1^ are assigned to the bending of Si−O−Si and Si−OH for SiO_2_, respectively. The appearance of the peaks at 3430 and 1630 cm^−1^ could be attributed to adsorbed water and silanol groups of both SiO_2_ and MCM−41. The FTIR spectrum of **SnP@MCM−41** shows the characteristic band of Si−O−Si stretching at 1055 cm^−1^. On the other hand, the absorption peak at 1070 cm^−1^ is assigned to the Si−O−Si stretching of **SnP@SiO_2_**. All other peaks are unchanged or slightly changed from those of the starting components. These observations indicate the attachment of **SnP** to MCM−41 and SiO_2_ to form **SnP@MCM−41** and **SnP@SiO_2_**, respectively.

The structural patterns of **SnP**, **SnP@MCM−41**, and **SnP@SiO_2_** were examined by X-ray diffractometer (Figure S1). It is evident from Figure S1 that the peaks are broad, and the XRD patterns are very similar for a typical amorphous solid. **SnP** shows two peaks centered at 9.8° and 17.8°. On the other hand, **SnP@MCM−41** and **SnP@SiO_2_** exhibit main peaks at 21.9° and 21.3°, respectively.

**Figure 2:**
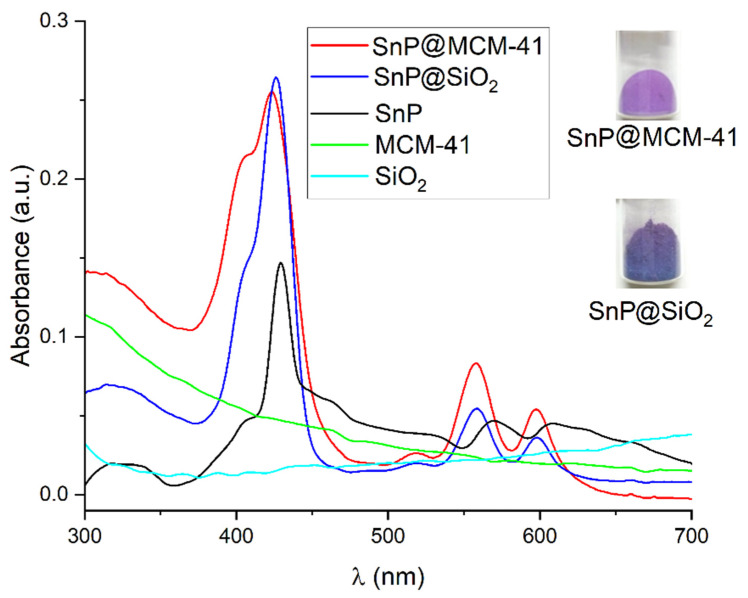
Solid-state UV–vis absorption spectra of **SnP**, SiO_2_, MCM−41, **SnP@MCM−41**, and **SnP@SiO_2_**. A Nujol paste was used for sample preparation. The photos represent both hybrid composite materials.

Solid-state UV–vis spectra reflect the light absorption properties of **SnP**, SiO_2_, MCM−41, **SnP@MCM−41**, and **SnP@SiO_2_** (Figure 2). **SnP** exhibits strong light absorption at 429 nm belonging to the Soret band and two weak absorptions at 569 and 607 nm corresponding to the Q−bands. In contrast, SiO_2_ and MCM−41 did not exhibit strong light absorbance in the visible region. Compared to **SnP**, **SnP@MCM−41** exhibits strong light absorption at 423 nm attributed to the Soret band and three weak bands at 519, 557, and 597 nm attributed to the Q−bands. Similarly, **SnP@SiO_2_** exhibits strong light absorption at 425 nm attributed to the Soret band and three weak bands at 519, 558, and 598 nm attributed to the Q−bands. This observation implies that the Soret band, as well as the Q−bands of **SnP**, were blue-shifted, which confirms the strong attachment of **SnP** in both hybrid composites, **SnP@MCM−41** and **SnP@SiO_2_**. The bandgap energy (*E*_g_) calculated by the Tauc plot method is 2.53 eV for **SnP@MCM−41**, which is lower than that of **SnP** (2.85 eV). Similarly, the bandgap energy of **SnP@SiO_2_** (2.64 eV) is lower than that of **SnP** (Table S2). Therefore, the enhanced light absorptions and narrower bandgaps of **SnP@MCM−41** and **SnP@SiO_2_** can effectively improve solar energy utilization, generating more photo-generated carriers to participate in the photocatalytic degradation reaction.

**Figure 3:**
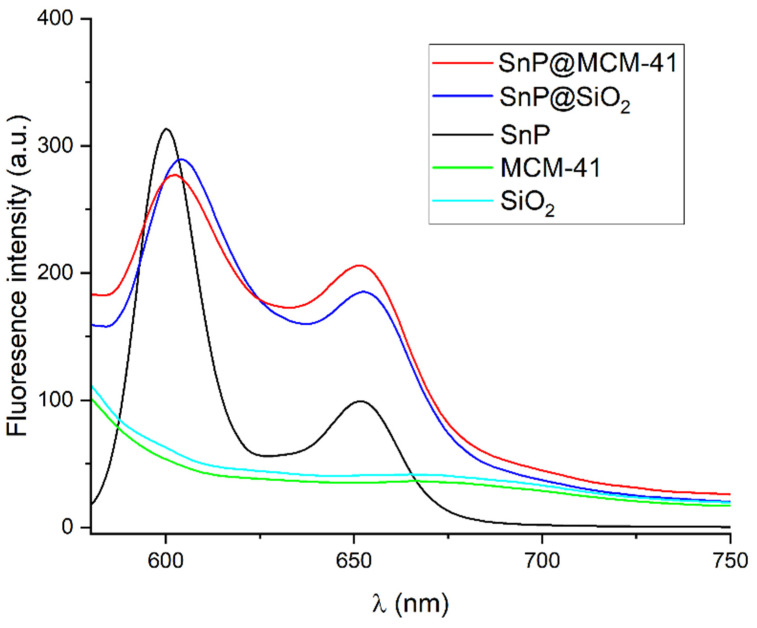
Fluorescence spectra of **SnP**, SiO_2_, MCM−41, **SnP@MCM−41**, and **SnP@SiO_2_** in a Nujol paste (*λ*_ex_ = 560 nm).

As the separation efficiency of photo-generated carriers has an important role in the photocatalytic process, its behavior was investigated by fluorescence spectroscopy. As shown in Figure 3, **SnP** emits fluorescence at 600 and 652 nm under excitation light with a wavelength of 560 nm, whereas SiO_2_ and MCM−41 do not exhibit fluorescence emission. Both hybrid composites similarly exhibited a set of emission bands, at 602 and 652 nm for **SnP@MCM−41** and at 603 and 653 nm for **SnP@SiO_2_**. The emission peak-to-peak ratio of **SnP** was changed for **SnP@MCM−41** and **SnP@SiO_2_**, which suggests strong adhesion.

**Figure 4:**
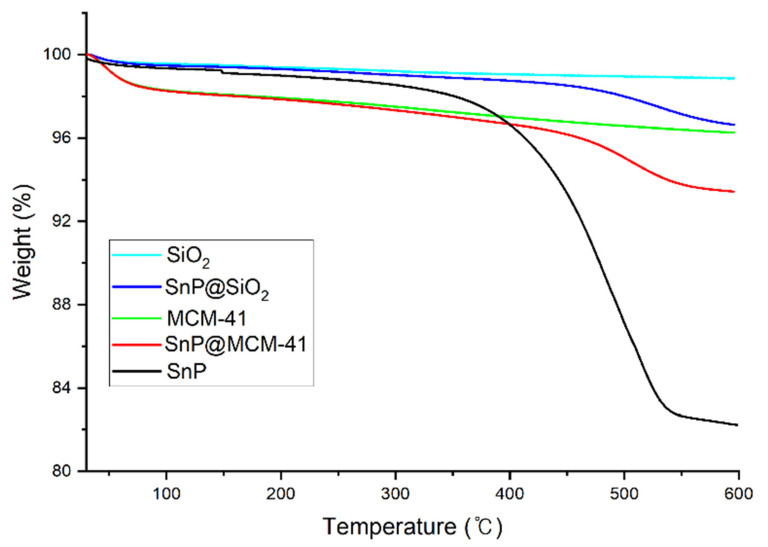
TGA curves of **SnP**, SiO_2_, MCM−41, **SnP@MCM−41**, and **SnP@SiO_2_**.

TGA curves for the materials are presented in Figure 4. **SnP** exhibits large weight loss starting at 350 °C. For **SnP@MCM−41**, the first change in the curve between 60 and 90 °C is assigned to the removal of physically adsorbed water on the surface, corresponding to a weight loss of approximately 2%. Additional weight loss was observed around 510 °C, attributed to the elimination of surface-bound organic groups of SnP. On the other hand, **SnP@SiO_2_** experienced apparent weight loss starting at 525 °C, where the surface-bound organic groups of SnP were removed. The TGA observations indicate that **SnP@MCM−41** and **SnP@SiO_2_** are thermally stable up to 550 °C.

**Figure 5:**
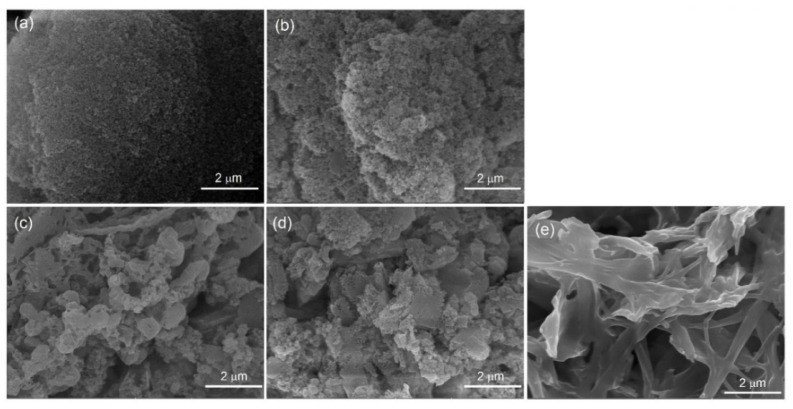
FE−SEM images of (**a**) SiO_2_, (**b**) **SnP@SiO_2_**, (**c**) MCM−41, (**d**) **SnP@MCM−41**, and (**e**) **SnP**.

The structural and morphological development of the fabricated **SnP@MCM−41** and **SnP@SiO_2_** was investigated by FE−SEM. The morphologies of **SnP@MCM−41** and **SnP@SiO_2_** along with those of the starting materials are presented in Figure 5 and Figure S2. The FE−SEM images show that **SnP@MCM−41** has an average diameter of approximately 100 nm with a narrow size distribution (Figure 5d). Compared to MCM−41, **SnP@MCM−41** has an irregular surface, possibly due to the chemical adsorption of **SnP** on the surface of MCM−41. On the other hand, the sphere-shaped **SnP@SiO_2_** has an average diameter of approximately 70 nm. Notably, the morphology of **SnP** does not exhibit a characteristic shape and size. EDS mapping images of **SnP@SiO_2_** and **SnP@MCM−41** (Figures S3 and S4) confirm the homogeneous distribution of elements (C, N, O, Si, and Sn). 

**Figure 6:**
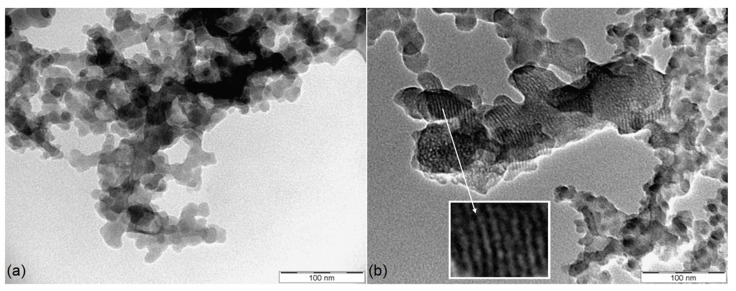
TEM images of (**a**) **SnP@SiO_2_** and (**b**) **SnP@MCM−41**. The inset represents an enlarged image of regular arrays of cylindrical mesopores.

The mesoporous structure of **SnP@MCM−41** was further confirmed by TEM. The TEM image of **SnP@MCM−41** (Figure 6) shows regular arrays of cylindrical mesopores forming a one-dimensional pore system in contrast to **SnP@SiO_2_**. The mesoporous channel of MCM−41 was thus expected to facilitate the efficient transport of electrons and holes photo-generated from **SnP**. The hydrodynamic particle sizes of **SnP**, SiO_2_, MCM−41, **SnP@MCM−41**, and **SnP@SiO_2_** in a suspension were measured by DLS. The average particle size in each aqueous solution had a narrow distribution with diameters of 40, 45, 70, 125, and 100 nm for **SnP**, SiO_2_, MCM−41, **SnP@MCM−41**, and **SnP@SiO_2_**, respectively (Figure S5).

The nitrogen sorption isotherms for determination of the permanent porosities of MCM−41 and **SnP@MCM−41** are presented in Table S3. The BET surface area and average pore size of **SnP@MCM−41** are 648.7 m^2^/g and 1.53 nm, respectively, suitable for catalytic applications due to the large available surface. The decreases in the surface area and pore size from the bare MCM−41 to **SnP@MCM−41** provide evidence for the incorporation of **SnP** within the channels of mesoporous frameworks. Therefore, MCM−41 was successfully coupled with **SnP** to form the **SnP@MCM−41** mesoporous hybrid composite that maintained high thermal stability and a large surface area.

### 2.2. Photocatalytic Degradation of Organic Dyes

**Figure 7:**
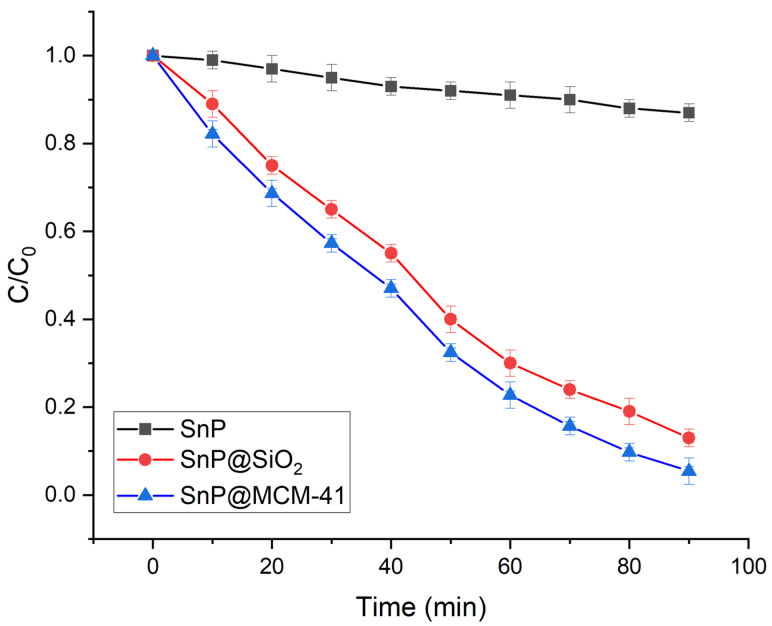
Photocatalytic degradation of the EG dye in an aqueous solution (pH = 7, T = 298 K) under visible light irradiation in the presence of **SnP**, **SnP@MCM−41**, and **SnP@SiO_2_**.

The photocatalytic performances of **SnP@MCM−41** and **SnP@SiO_2_** were investigated by the degradation of the organic dyes under visible light irradiation in aqueous solutions. For this purpose, anionic EG, cationic RhB, and neutral MCP were selected as target pollutant dyes for photocatalytic degradation. After approximately 30 min to reach the adsorption–desorption equilibrium, 5, 18, and 25% of EG was adsorbed by **SnP**, **SnP@SiO_2_**, and **SnP@MCM−41**, respectively (Figure S6). This indicates that the large surface area and mesoporous framework of **SnP@MCM−41** can promote mass diffusion and enhance adsorption. Time-dependent absorption spectra of EG in the presence of **SnP@MCM−41** under visible light irradiation are shown in Figure S7. Negligible decay of the EG dye was observed in the absence of either visible light or photocatalyst **SnP@MCM−41** (Figure S8), which implies that visible light is essential for the degradation of the EG dye in addition to photocatalysts. In Figure S7, the absorbance of the EG dye at 629 nm decreases with the increase in the visible light irradiation time. Figure 7 shows that all prepared catalysts exhibit significant progress in the photodegradation of EG in an aqueous solution.

The degradation of the EG dye in the presence of photocatalysts can be described by its degradation efficiency, (C_0_ − C)/C_0_, where C_0_ is the initial concentration of EG and C is the concentration at time *t*. The observed degradation rate of EG is 13% for **SnP**, 87% for **SnP@SiO_2_**, and 95% for **SnP@MCM−41** within 90 min of irradiation of visible light (Figure 7). Therefore, the photocatalyst **SnP@MCM−41** exhibits better performance toward the degradation of the EG dye than **SnP** and **SnP@SiO_2_**. To further elucidate the reaction kinetics for the decay of the EG dye, we used the pseudo-first-order concept, expressed by ln(C_0_/C) = k*t*, which is generally used for a photocatalytic degradation experiment if the initial concentration of the dye is low, where k is the pseudo-first-order degradation rate constant. Based on the data plotted in Figure 7, the reaction kinetics of EG dye degradation are presented in Figure S9. The first-order rate constant for the degradation of the EG dye is 0.002 min^−1^ for **SnP**, 0.022 min^−1^ for **SnP@SiO_2_**, and 0.029 min^−1^ for **SnP@MCM−41**. 

**Figure 8:**
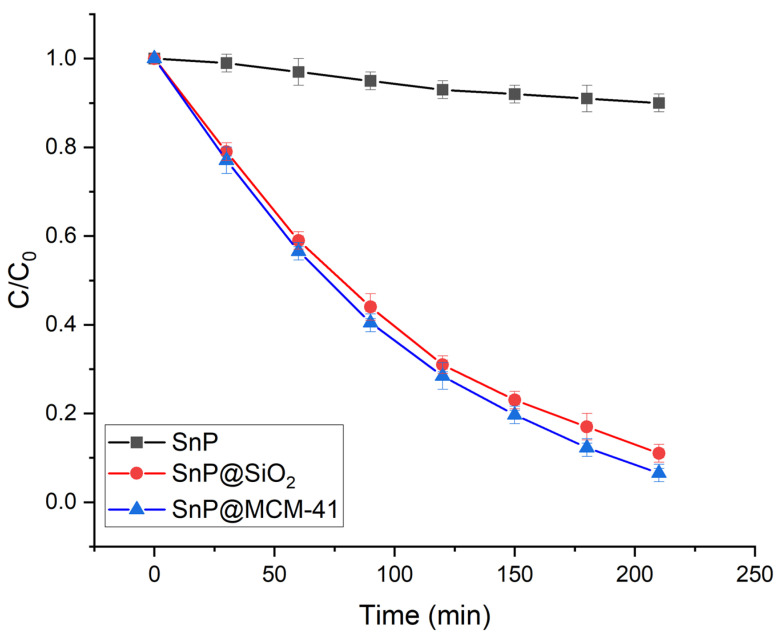
Photocatalytic degradation of the RhB dye in an aqueous solution (pH = 7, T = 298 K) under visible light irradiation.

The photodegradation performances of **SnP@MCM−41** and **SnP@SiO_2_** were further analyzed toward diverse targets (cationic RhB and neutral MCP dyes) under visible light irradiation. Time-dependent absorption spectra of RhB in the presence of **SnP@MCM−41** under visible light irradiation are shown in Figure S10. As shown in Figure 8 and Figure S11, the pseudo-first-order rate constant for the degradation of RhB is 0.0006 min^−1^ for **SnP**, 0.011 min^−1^ for **SnP@SiO_2_**, and 0.013 min^−1^ for **SnP@MCM−41**.

**Figure 9:**
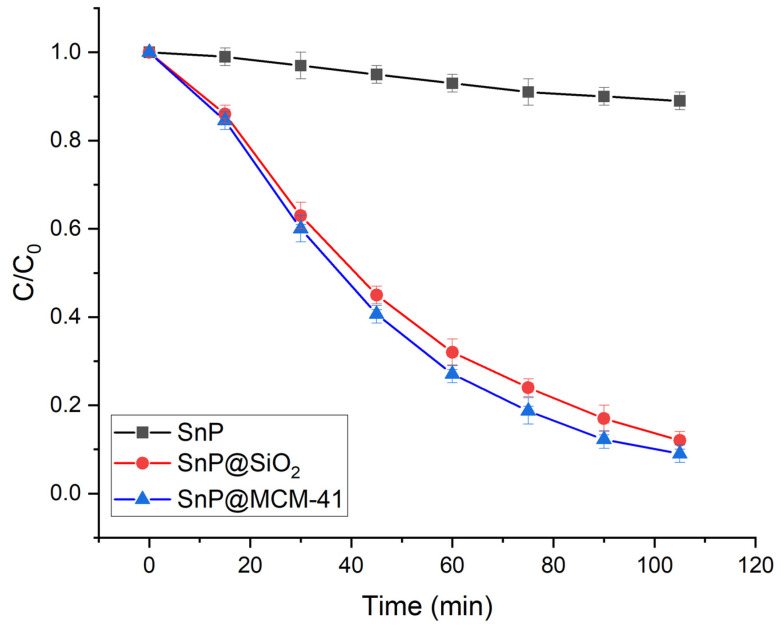
Photocatalytic degradation of the MCP dye in an aqueous solution (pH = 7, T = 298 K) under visible light irradiation.

On the other hand, time-dependent absorption spectra of MCP in the presence of the photocatalyst **SnP@MCM−41** under visible light irradiation are shown in Figure S12. The pseudo-first-order rate constant for the degradation of MCP is 0.001 min^−1^ for **SnP**, 0.021 min^−1^ for **SnP@SiO_2_**, and 0.024 min^−1^ for **SnP@MCM−41** (Figure 9 and Figure S13).

The reusability of the photocatalysts **SnP@MCM−41** and **SnP@SiO_2_** is crucial for practical application, which was evaluated by recycling tests on **SnP@MCM−41** toward EG dye degradation (Figure S14). Even after 10 consecutive cycles, **SnP@MCM−41** still maintains a high performance for the degradation of the EG dye with a reduction of only 8%, which indicates that the photocatalyst **SnP@MCM−41** has remarkable stability. After the degradation reaction, the structure of **SnP@MCM−41** was additionally analyzed to confirm the stability of this photocatalyst. As shown in Figures S15 and S16, the FTIR spectrum and FE−SEM image of the used **SnP@MCM−41** closely resembles that of the as-prepared state, which indicates that the mesoporous framework of this photocatalyst was intact during the photocatalytic reaction. Moreover, the process for the recovery of these photocatalysts, **SnP@MCM−41** and **SnP@SiO_2_**, from the reaction mixture was very simple through successive filtration, washing, and drying procedures.

We optimized the reaction conditions in terms of the EG dye/photocatalyst ratio, temperature, and pH of the solution. Photodegradation experiments were carried out at different temperatures to evaluate the effect of temperature on the degradation of the EG dye by the photocatalyst **SnP@MCM−41**. With the increase in temperature, the degradation efficiency increases up to 50 °C (Figure S17). The pH of the aqueous EG dye solution apparently affected the degradation rate of the EG dye, as shown in Figure S18. The rate of degradation increases from pH = 2 to pH = 8, and then decreases to pH = 12. To evaluate the effect of the dye/photocatalyst ratio on the degradation of the EG dye, various concentrations of the EG solution (5, 10, 15, 20, 25, 30, 35, and 40 mg L^−1^) were applied with a constant amount (20 mg) of the photocatalyst **SnP@MCM−41**. The degradation rate decreases with the increase in the concentration of the EG dye. Most of the EG dye is degraded at concentrations of 5−25 mg L^−1^, while approximately 60% of the dye is degraded even at 40 mg L^−1^ (Figure S19).

Among all photocatalysts, **SnP@MCM−41** shows the highest photodegradation rate, which can remove about 95% of EG dye within 90 min. To determine the effect of the amount of **SnP** adsorbed onto **SnP@MCM−41** for photocatalytic activity, a series of **SnP@MCM−41** samples varying only in a molar amount of **SnP** with respect to MCM−41 per gram (MCM−41/g) were used for the measurement of the EG dye degradation rate (Figure S20). The photodegradation rate of EG for composites **SnP@MCM−41** (0.01 mmol/g to 0.2 mmol/g) was higher than pure **SnP**. The rate also increased when the molar amount of **SnP** compared to MCM−41/g increased in the composite, reaching the maximum of 0.1 mmol/g. After that, the rates decreased from 0.15 mmol/g to 0.2 mmol/g. When the molar amount of **SnP** to MCM−41/g is lower than 0.05 mmol/g, the host photocatalyst **SnP** has low content in the composite to reduce the amount of photogenerated carriers, resulting in relatively low catalytic efficiency. When the molar amount ratio is higher than 0.1 mmol/g, **SnP** significantly agglomerates on the MCM−41 surface, reducing reactive sites, suppressing photogenerated charge separation, and gradually reducing degradation efficiency of EG dye. This suggests the synergistic effect between **SnP** and MCM−41 is responsible for the noticeably enhanced photocatalytic activity of the composite **SnP@MCM−41**.

We previously reported a possible mechanism for the photodegradation of organic dyes by porphyrin-based nanomaterials in an aqueous solution under visible light irradiation [38,39]. Usually, the mechanism includes five steps. In step 1, a photocatalyst in an aqueous solution absorbs light upon exposure to visible light irradiation along with the EG dye. Valence-band electrons are promoted to the conduction band after crossing the bandgap. This assists in the generation of electron–hole (e^−^/*h*^+^) pairs at the surface of the photocatalyst. These photogenerated holes (*h*^+^) react with H_2_O to form a highly reactive hydroxyl radical (^•^OH) (step 2). On the other hand, the excited electron reacts with the dissolved O_2_ to generate highly reactive superoxide radical anions (O_2_^−•^) in step 3. These photogenerated, highly reactive superoxide radical anions and hydroxyl radicals react with the EG dye and degrade it into small molecules and finally to CO_2_ and H_2_O (steps 4 and 5). The photocatalytic degradation mechanism of 4-chlorophenol and acid orange 7 by SnP-based photocatalysts was similarly discussed [14].

The mechanism consists of five steps for a porphyrin-based porous framework **P**:

**P** + *hν* → **P^∗^** ( e^−^ + *h*^+^) (1)

H_2_O + *h*^+^ → ^•^OH + H^+^ (2)

O_2_ + 2e^−^ → O_2_^−•^ (3)

^•^OH + EG → degraded products (4)

O_2_^−•^ + EG → degraded products (5)

Radical trapping experiments were carried out to detect the photogenerated reactive species during the photocatalytic degradation of the EG dye [44,45]. We used ethylenediaminetetraacetic acid disodium (Na_2_EDTA) to capture holes, *tert*-butanol (*^t^*BuOH) to capture hydroxyl radicals (^•^OH), and *para*-benzoquinone (*p*-BQ) for superoxide radical anions (O_2_^−•^) during the photodegradation experiment of the EG dye in the presence of **SnP@MCM−41**. Figure S8 confirms that the EG dye degradation rate is critically affected in the presence of *^t^*BuOH as well as *p*-BQ. Superoxide radicals (O_2_^−•^) are a major reactive species compared to hydroxyl radicals (^•^OH) responsible for the catalytic degradation of the EG dye in an aqueous solution. However, the degradation of the EG dye was not affected by the presence of Na_2_EDTA or photogenerated holes.

**Figure 10:**
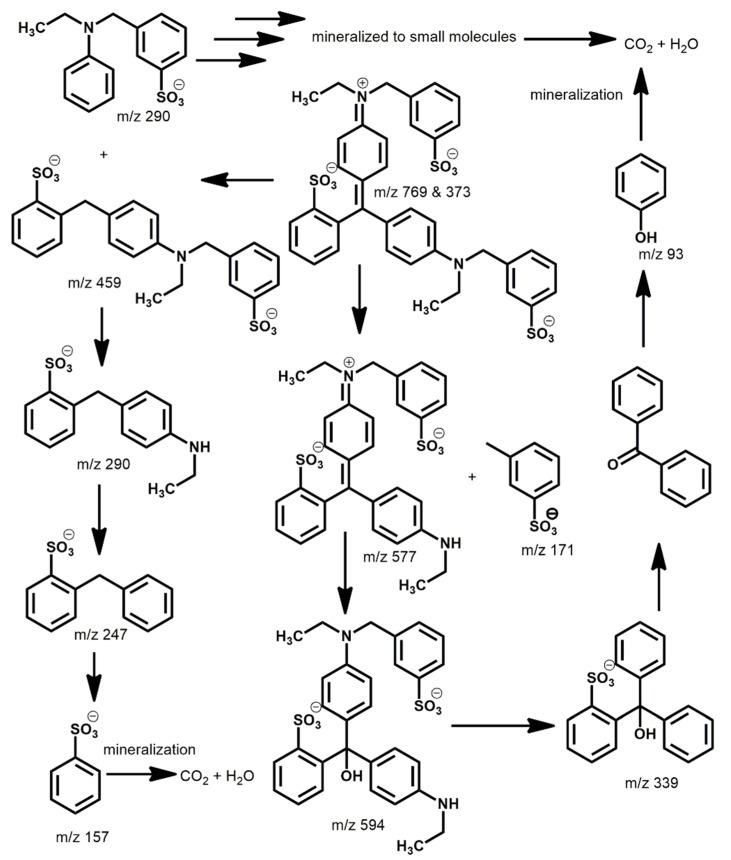
Possible intermediates for the degradation of the EG dye in the presence of the photocatalyst **SnP@MCM−41**.

We also analyzed the degradation products of the EG dye after visible light irradiation in the presence of the photocatalyst **SnP@MCM−41**. The reaction mixture was analyzed by electrospray ionization–mass spectrometry (ESI-MS) after 45 min for each photodegradation experiment (Figure S21). New peaks in the mass spectra confirmed the degradation of the EG dye to new small molecules [46]. Based on the mass spectra in Figure S16, possible intermediates for the degradation of the EG dye are shown in Figure 10. Initially, the base peak (*m*/*z* = 769 for [EG−Na]) and another peak (*m*/*z* = 373 for [EG−2Na]) belong to the anionic EG dye. The EG dye can be fragmented in two different manners. The first is through cleavage of the tertiary amine bond and formation of two low-molecular-weight fragments with a secondary amine (*m*/*z* = 577) and 3-methyl-benzenesulfonic acid (*m*/*z* = 171). This secondary amine can further undergo hydrolysis and generate a tertiary alcohol derivative (*m*/*z* = 594). Again, successive oxidation of tertiary alcohol leads to formation of lower-molecular-weight fragments (*m*/*z* = 339 and 93). In the second process, the cleavage of the C–C double bond leads to formation of two lower-molecular-weight amine fragments (*m*/*z* = 459 and 290). These low-molecular-weight amines can then undergo successive hydrolysis that leads to formation of benzenesulfonic acid (*m*/*z* = 157). Finally, these lower-molecular-weight intermediate molecules were further fragmented and mineralized into CO_2_ and H_2_O. The total organic carbon (TOC) value was calculated to estimate the removal of the EG dye by photocatalysts [47]. The TOC removal percentage attained using **SnP@MCM−41** was only 75%.

Finally, the degradation performances of organic dyes represented by the pseudo-first-order rate constants for **SnP@MCM−41** and **SnP@SiO_2_** were compared to those of selected photocatalysts reported in the literature. As listed in Table 1, **SnP@MCM−41** and **SnP@SiO_2_** under visible light irradiation exhibit higher than or comparable degradation performances for EG, MCP, and RhB to those of most reported photocatalysts.

## 3. Materials and Methods

MCM−41 and SiO_2_ nanoparticles were purchased from Sigma–Aldrich. All other chemicals were purchased from TCI and used without further purification unless otherwise specified. (*trans*-Dihydroxo)(5,10,15,20-tetraphenylporphyrinato)tin(IV) (SnP) was prepared similarly according to the previously reported procedure [59]. CH_2_Cl_2_ and pyrrole were distilled from a solution over calcium hydride. Fourier-transform infrared (FTIR) spectra were acquired using a Shimadzu FTIR−8400S spectrophotometer (Shimadzu, Tokyo, Japan). Steady-state ultraviolet–visible (UV–vis) spectra were recorded using a Shimadzu UV-3600 spectrophotometer (Shimadzu, Tokyo, Japan). Fluorescence spectra were recorded using a Shimadzu RF-5301PC fluorescence spectrophotometer (Shimadzu, Tokyo, Japan). Powder X-ray diffraction (PXRD) patterns were obtained on a Bruker AXS D8 Advance powder X-ray diffractometer (Bruker, Billerica, MA, USA). A thermogravimetric analysis (TGA) was performed using an Auto-TGA Q500 instrument (TA Instruments, New Castle, DE, USA). The Brunauer−Emmett−Teller (BET) surface area was determined using an analyzer (BELSORP-mini volumetric adsorption equipment) through N_2_ adsorption isotherms at 77 K. The surface area and pore size were estimated using an Autosorb-iQ and Quadrasorb SI. Field-emission scanning electron microscopy (FE−SEM) and transmission electron microscopy (TEM) images were acquired using a MAIA III (TESCAN, Brno, Czech Republic) and JEOL/JEM 2100, respectively. Zeta potentials were measured with an Otsuka Electronics ELSZ−2. Dynamic light scattering (DLS) experiments and inductively coupled plasma (ICP) analysis were performed using a NanoBrook 90Plus DLS size analyzer (Brookhaven, NY, USA) and ICP−Spectrociros CCD instrument, respectively.

### 3.1. Fabrication of **SnP@MCM−41**

Dried MCM−41 (3.0 g) was added into a solution of **SnP** (0.23 g) dissolved in CH_2_Cl_2_ (100 mL, ~3.0 mM) and stirred for 24 h at room temperature. The solids were filtered and washed three times successively with acetone, CH_2_Cl_2_, and *N*,*N*−dimethylformamide. Afterward, the solids were dried in a vacuum oven for 4 h at 80 °C, which yielded a powder of **SnP@MCM−41** (2.864 g).

### 3.2. Fabrication of **SnP@SiO_2_**

Using dried SiO_2_ (3.0 g), a powder of **SnP@SiO_2_** (3.003 g) was obtained by the same procedure.

### 3.3. Photocatalytic Degradation Experiment

The photocatalytic efficiencies of these hybrid composites were investigated by photodegrading EG, RhB, and MCP dyes in each aqueous solution. In a typical procedure, 20 mg of the photocatalyst was added to 250 mL of an aqueous solution of EG (20 mg L^−1^, distilled water with pH of 7.0) with stirring at 298 K. The reaction mixture was allowed to stand in dark for 30 min to reach the adsorption−desorption equilibrium. Afterward, the irradiation process was started using a 150 W xenon arc lamp with a UV cut-off filter (ABET Technologies, Old Gate Lane, Milford, CT, USA) at 298 K. After irradiation with visible light, a 3 mL suspension was collected at regular intervals. The photocatalyst was separated from the solution by centrifugation and collected by filtration with filter paper. The concentration of EG was determined by measuring the absorbance at 629 nm using a UV–vis spectrophotometer. Similarly, an aqueous solution of RhB (40 mg L^−1^) dye was prepared, and its concentration was determined by assessing the absorbance at 553 nm. For the MCP dye, we used an aqueous solution of MCP (65 mg L^−1^) and determined its concentration at 435 nm.

## 4. Conclusions

Hybrid composites of **SnP@MCM−41** and **SnP@SiO_2_** were fabricated by the reaction of (*trans*-dihydroxo)(5,10,15,20-tetraphenylporphyrinato)tin(IV) (**SnP**) with the mesostructured MCM−41 and nanoparticle SiO_2_, respectively. The formation of hybrid composites was readily achieved through condensation between the hydroxo ligand of **SnP** and silanol groups of MCM−41 or SiO_2_. These hybrid composites were fully characterized by FTIR spectroscopy, UV–vis spectroscopy, fluorescence spectroscopy, TEM, and FE−SEM techniques. The incorporated **SnP** onto the mesoporous structure provided efficient photocatalytic degradation for the anionic EG, cationic RhB, and neutral MCP dyes under visible light irradiation in an aqueous solution. The pseudo-first-order degradation rate constant of organic dyes for **SnP@MCM−41** was higher than those of **SnP@SiO_2_** and **SnP**. These visible light photocatalysts showed remarkable stability and reliable reusability. Our report on visible light photocatalysts provides a valuable contribution for the treatment of dyed wastewater and encourages the development of highly effective hybrid composites composed of porous materials and visible light photocatalysts for applications in environmental remediation.

## Figures and Tables

**Table 1:** Performances of degradation for EG, MCP, and RhB by various photocatalysts.

Photocatalyst	Dye	Rate Constant (min^−1^)	References
TiO_2_ (P−25)	EG	0.0473	[48]
γ−Irradiation	EG	0.0018	[49]
TiO_2_ (UV)	EG	0.025	[50]
CdS (one-dimensional)	EG	0.008	[51]
**SnP**	EG	0.002	This study
**SnP@SiO_2_**	EG	0.022	This study
**SnP@MCM−41**	EG	0.029	This study
Electrochemical-oxidation	MCP	0.32	[52]
ZnO	MCP	0.022	[53]
**SnP**	MCP	0.001	This study
**SnP@SiO_2_**	MCP	0.021	This study
**SnP@MCM−41**	MCP	0.024	This study
TiO_2_ (P−25)	RhB	0.001	[53]
Co_0.6_Zn_0.4_Fe_2_O_4_	RhB	0.015	[54]
TiO_2_/MgZnAl	RhB	0.005	[55]
CdS	RhB	0.003	[56]
ZnO	RhB	0.009	[57]
TiO_2_ NTs/Bi_2_MoO_6_	RhB	0.0077	[58]
**SnP**	RhB	0.0006	This study
**SnP@SiO_2_**	RhB	0.011	This study
**SnP@MCM−41**	RhB	0.013	This study

## Data Availability

Not applicable.

## Supplementary Materials

The following supporting information can be downloaded at: https://www.mdpi.com/article/10.3390/molecules28041886/s1, Table S1: Zeta potentials and mobilities of SiO_2_, **SnP@SiO_2_**, MCM−41, **SnP@MCM−41**, and **SnP**; Table S2: Band-gap energies (*E*_g_) calculated by the Tauc plot method; Table S3: Porosities of MCM-41 and **SnP@MCM−41**; Figure S1: XRD patterns of **SnP**, **SnP@SiO_2_**, and **SnP@MCM−41**; Figure S2: High-resolution FE−SEM images of (a) SiO_2_, (b) **SnP@SiO_2_**, (c) MCM−41, (d) **SnP@MCM−41**, and (e) **SnP**; Figure S3: EDS mapping of **SnP@SiO_2_**; Figure S4: EDS mapping of **SnP@MCM−41**; Figure S5: Hydrodynamic particle size distributions of **SnP**, SiO_2_, MCM−41, **SnP@MCM−41**, and **SnP@SiO_2_** obtained by the DLS method; Figure S6: Adsorptivities of **SnP**, **SnP@MCM−41**, and **SnP@SiO_2_** for the EG dye; Figure S7: Time-dependent absorption spectra of EG in the presence of **SnP@MCM−41** under visible light irradiation; Figure S8: Various external effects on the degradation of the EG dye in the presence of **SnP@MCM−41** under visible light irradiation. ([Na_2_EDTA]_0_ = [*p*-BQ]_0_ = [*^t^*BuOH]_0_ = 2 mM, pH = 7.0, T = 298 K); Figure S9: Kinetics for the photocatalytic degradation of EG under visible light irradiation by the photocatalysts **SnP**, **SnP@MCM−41**, and **SnP@SiO_2_**; Figure S10: Time-dependent absorption spectra of the RhB dye in the presence of **SnP@MCM−41** under visible light irradiation; Figure S11: Kinetics for the photocatalytic degradation of RhB under visible light irradiation by the photocatalysts **SnP**, **SnP@MCM−41**, and **SnP@SiO_2_**; Figure S12: Time-dependent absorption spectra of the MCP dye in the presence of **SnP@MCM−41** under visible light irradiation; Figure S13: Kinetics for the photocatalytic degradation of MCP under visible light irradiation by the photocatalysts **SnP**, **SnP@MCM−41**, and **SnP@SiO_2_**; Figure S14: Typical catalytic cycle (up to 10 cycles) for the photocatalyst **SnP@MCM−41** for the degradation of the EG dye; Figure S15: Comparison of FT-IR spectra of **SnP@MCM−41** before and after the degradation of the EG dye; Figure S16: Comparison of FE−SEM images of SnP@MCM−41 before and after the degradation of EG dye; Figure S17: Effect of the temperature on the degradation of the EG dye by **SnP@MCM−41**; Figure S18: Effect of the pH of the solution of the EG dye for photodegradation by **SnP@MCM−41**; Figure S19: Effect of the concentration of the EG dye for photodegradation by **SnP@MCM−41** (20 mg) within 90 min of visible light irradiation; Figure S20: Effect of the amount of **SnP** adsorbed onto **SnP@MCM−41** composite for photocatalytic degradation of EG dye, where X = mmol of **SnP** per gram of MCM−41; Figure S21: ESI-MS spectrum (negative ion mode) of the reaction mixture of EG in the presence of **SnP@MCM−41** after 45 min of visible light irradiation.

## References

[1] Schwarzenbach R.P., Egli T., Hofstetter T., Von Gunten U., Wehrli B. (2010). Global Water Pollution and Human Health. Annu. Rev. Environ. Resour..

[2] Naidu R., Biswas B., Willett I.R., Cribb J., Kumar Singh B., Paul Nathanail C., Coulon F., Semple K.T., Jones K.C., Barclay A. (2021). Chemical Pollution: A Growing Peril and Potential Catastrophic Risk to Humanity. Environ. Int..

[3] Wang Q., Li H., Yu X., Jia Y., Chang Y., Gao S. (2020). Morphology regulated Bi_2_WO_6_ nanoparticles on TiO_2_ nanotubes by solvothermal Sb^3+^ doping as effective photocatalysts for wastewater treatment. Electrochim. Acta.

[4] Parvulescu V.I., Epron F., Garcia H., Granger P. (2022). Recent Progress and Prospects in Catalytic Water Treatment. Chem. Rev..

[5] Ain Q.U., Rasheed U., Yaseen M., Zhang H., Tong Z. (2020). Superior dye degradation and adsorption capability of polydopamine modified Fe_3_O_4_-pillared bentonite composite. J. Hazard. Mater..

[6] Glugoski L.P., de Jesus Cubas P., Fujiwara S.T. (2017). Reactive Black 5 dye degradation using filters of smuggled cigarette modified with Fe^3+^. Environ. Sci. Pollut. Res..

[7] Balcha A., Yadav O.P., Dey T. (2016). Photocatalytic degradation of methylene blue dye by zinc oxide nanoparticles obtained from precipitation and sol-gel methods. Environ. Sci. Pollut. Res..

[8] Garrido-Cardenas J.A., Esteban-García B., Agüera A., Sánchez-Pérez J.A., Manzano-Agugliaro F. (2020). Wastewater Treatment by Advanced Oxidation Process and Their Worldwide Research Trends. Int. J. Environ. Res. Public Health.

[9] Dewil R., Mantzavinos D., Poulios I., Rodrigo M.A. (2017). New perspectives for Advanced Oxidation Processes. J. Environ. Manag..

[10] O’Neill J.S., Kearney L., Brandon M.P., Pryce M.T. (2022). Design Components of Porphyrin-Based Photocatalytic Hydrogen Evolution Systems: A Review. Coord. Chem. Rev..

[11] Mota H.P., Quadrado R.F.N., Iglesias B.A., Fajardo A.R. (2020). Enhanced Photocatalytic Degradation of Organic Pollutants Mediated by Zn(II)-Porphyrin/Poly(Acrylic Acid) Hybrid Microparticles. Appl. Catal. B Environ..

[12] Li Y., Wang L., Gao Y., Yang W., Li Y., Guo C. (2018). Porous metalloporphyrinic nanospheres constructed from metal 5,10,15,20-tetraksi(4′-ethynylphenyl)porphyrin for efficient catalytic degradation of organic dyes. RSC Adv..

[13] Jang J.H., Jeon K.-S., Oh S., Kim H.-J., Asahi T., Masuhara H., Yoon M. (2007). Synthesis of Sn-Porphyrin-Intercalated Trititanate Nanofibers:  Optoelectronic Properties and Photocatalytic Activities. Chem. Mater..

[14] Kim W., Park J., Jo H.J., Kim H.-J., Choi W. (2008). Visible Light Photocatalysts Based on Homogeneous and Heterogenized Tin Porphyrins. J. Phys. Chem. C.

[15] Guo X., Li Y.-Y., Shen D.-H., Song Y.-Y., Wang X., Liu Z.-G. (2013). Immobilization of cobalt porphyrin on CeO_2_@SiO_2_ core-shell nanoparticles as a novel catalyst for selective oxidation of diphenylmethane. J. Mol. Catal. A Chem..

[16] Yoo H.-Y., Yan S., Ra J.W., Jeon D., Goh B., Kim T.-Y., Mackeyev Y., Ahn Y.-Y., Kim H.-J., Wilson L.J. (2016). Tin porphyrin immobilization significantly enhances visible-light-photosensitized degradation of Microcystins: Mechanistic implications. Appl. Catal. B Environ..

[17] Wang J., Zhong Y., Wang X., Yang W., Bai F., Zhang B., Alarid L., Bian K., Fan H. (2017). pH-Dependent Assembly of Porphyrin-Silica Nanocomposites and Their Application in Targeted Photodynamic Therapy. Nano Lett..

[18] Kresge C.T., Leonowicz M.E., Roth W.J., Vartuli J.C., Beck J.S. (1992). Ordered mesoporous molecular sieves synthesized by a liquid-crystal template mechanism. Nature.

[19] Martínez-Edo G., Balmori A., Pontón I., Martí del Rio A., Sánchez-García D. (2018). Functionalized Ordered Mesoporous Silicas (MCM-41): Synthesis and Applications in Catalysis. Catalysts.

[20] Costa J.A.S., Jesus R.A., Santos D., Manoa J.F., Romao L.P.C., Paranhos C.M. (2020). Recent progresses in the adsorption of organic, inorganic, and gas compounds by MCM-41-based mesoporous materials. Microporous Mesoporous Mater..

[21] Kim H.J., Park K.-M., Ahn T.K., Kim S.K., Kim K.S., Kim D., Kim H.-J. (2004). Novel fullerene–porphyrin–fullerene triad linked by metal axial coordination: Synthesis, X-ray crystal structure, and spectroscopic characterizations of trans-bis([60]fullerenoacetato)tin(iv) porphyrin. Chem. Commun..

[22] Kim H.J., Jeon W.S., Lim J.H., Hong C.S., Kim H.-J. (2007). Synthesis, X-ray crystal structure, and electrochemistry of trans-bis(ferrocenecarboxylato)(tetraphenylporphyrinato)tin(IV). Polyhedron.

[23] Kim H.J., Jang J.H., Choi H., Lee T., Ko J., Yoon M., Kim H.-J. (2008). Photoregulated Fluorescence Switching in Axially Coordinated Tin(IV) Porphyrinic Dithienylethene. Inorg. Chem..

[24] Kim M.K., Shee N.K., Lee J., Yoon M., Kim H.-J. (2019). Photoinduced Electron Transfer upon Supramolecular Complexation of (Porphyrinato) Sn-Viologen with Cucurbit[7]uril. Photochem. Photobiol. Sci..

[25] Shee N.K., Kim M.K., Kim H.-J. (2019). Fluorescent chemosensing for aromatic compounds by supramolecular complex composed of tin(IV) porphyrin, viologen, and cucurbit[8]uril. Chem. Commun..

[26] Kim H.-J., Bampos N., Sanders J.K.M. (1999). Assembly of Dynamic Heterometallic Oligoporphyrins Using Cooperative Zinc-Nitrogen, Ruthenium-Nitrogen, and Tin-Oxygen Coordination. J. Am. Chem. Soc..

[27] Indelli M.T., Chiorboli C., Ghirotti M., Orlandi M., Scandola F., Kim H.J., Kim H.-J. (2010). Photoinduced Electron Transfer in Ruthenium(II)/Tin(IV) Multiporphyrin Arrays. J. Phys. Chem. B.

[28] Kim H.J., Shee N.K., Park K.-M., Kim H.-J. (2019). Assembly and X-ray crystal structures of heterometallic multiporphyrins with complementary coordination between ruthenium (II) and tin (IV) porphyrins. Inorg. Chim. Acta.

[29] Kim H.-J., Jo H.J., Kim J., Kim S.-Y., Kim D., Kim K. (2005). Supramolecular self-assembly of tin(IV) porphyrin channels stabilizing single-file chains of water molecules. CrystEngComm.

[30] Li C., Park K.-M., Kim H.-J. (2015). Ionic assembled hybrid nanoparticle consisting of tin(IV) porphyrin cations and polyoxomolybdate anions, and photocatalytic hydrogen production by its visible light sensitization. Inorg. Chem. Commun..

[31] Shee N.K., Lee C.-J., Kim H.-J. (2022). Octahedral Tin(IV) Porphyrin-Based Porous Square-Grid Coordination Networks Exhibiting Selective Uptake of CO_2_ over N_2_. Bull. Korean Chem. Soc..

[32] Kim S.-H., Kim H.-J. (2022). Photocatalytic Hydrogen Production by the Sensitization of Sn(IV)-Porphyrin Embedded in a Nafion Matrix Coated on TiO_2_. Molecules.

[33] Shee N.K., Kim M.K., Kim H.-J. (2020). Supramolecular Porphyrin Nanostructures Based on Coordination-Driven Self-Assembly and Their Visible Light Catalytic Degradation of Methylene Blue Dye. Nanomaterials.

[34] Shee N.K., Kim H.-J. (2021). Self-Assembled Nanomaterials Based on Complementary Sn(IV) and Zn(II)-Porphyrins, and Their Photocatalytic Degradation for Rhodamine B Dye. Molecules.

[35] Shee N.K., Kim H.-J. (2022). Three Isomeric Zn(II)-Sn(IV)-Zn(II) Porphyrin-Triad-Based Supramolecular Nanoarchitectures for the Morphology-Dependent Photocatalytic Degradation of Methyl Orange. ACS Omega.

[36] Shee N.K., Kim H.-J. (2022). Morphology-controlled self-assembled nanostructures of complementary metalloporphyrin triads through intermolecular coordination tuning and their photocatalytic degradation for Orange II. Inorg. Chem. Front..

[37] Shee N.K., Kim H.-J. (2022). Coordination framework materials fabricated by the self-assembly of Sn(IV) porphyrins with Ag(I) ions for the photocatalytic degradation of organic dyes in wastewater. Inorg. Chem. Front..

[38] Shee N.K., Kim H.-J. (2022). Sn(IV) Porphyrin-Based Ionic Self-Assembled Nanostructures and Their Application in Visible Light Photo-Degradation of Malachite Green. Catalysts.

[39] Shee N.K., Kim H.-J. (2022). Sn(IV)-Porphyrin-Based Nanostructures Featuring Pd(II)-Mediated Supramolecular Arrays and Their Photocatalytic Degradation of Acid Orange 7 Dye. Int. J. Mol. Sci..

[40] Shee N.K., Kim H.-J. (2022). Supramolecular squares of Sn(IV)porphyrins with Re(I)-corners for the fabrication of self-assembled nanostructures performing photocatalytic degradation of Eriochrome Black T dye. Inorg. Chem. Front..

[41] Jank M., Köser H., Lücking F., Martienssen M., Wittchen S. (1998). Decolorization and Degradation of Erioglaucine (Acid Blue 9) Dye in Wastewater. Environ. Technol..

[42] Larowska D., O’Brien J.M., Senge M.O., Burdzinski G., Marciniak B., Lewandowska-Andralojc A. (2020). Graphene Oxide Functionalized with Cationic Porphyrins as Materials for the Photodegradation of Rhodamine B. J. Phys. Chem. C.

[43] Khezrianjoo S., Revanasiddappa H.D. (2015). Effect of operational parameters and kinetic study on the photocatalytic degradation of m-cresol purple using irradiated ZnO in aqueous medium. Water Qual. Res. J..

[44] Liu D., Song J., Chung J.S., Hur S.H., Choi W.M. (2022). ZnO/Boron Nitride Quantum Dots Nanocomposites for the Enhanced Photocatalytic Degradation of Methylene Blue and Methyl Orange. Molecules.

[45] Rojas-García E., García-Martínez D.C., López-Medina R., Rubio-Marcos F., Castañeda-Ramírez A.A., Maubert-Franco A.M. (2022). Photocatalytic Degradation of Dyes Using Titania Nanoparticles Supported in Metal-Organic Materials Based on Iron. Molecules.

[46] Borhade A.V., Tope D.R., Dabhade G.B. (2017). Removal of erioglaucine dye from aqueous medium using ecofriendly synthesized ZnMnO_3_ photocatalyst. J. Surf. Sci. Nanotech..

[47] Wang Z., Xia L., Chen J., Ji L., Zhou Y., Wang Y., Cai L., Guo J., Song W. (2020). Fine Characterization of Natural SiO_2_-Doped Catalyst Derived from Mussel Shell with Potential Photocatalytic Performance for Organic Dyes. Catalysts.

[48] Daneshvar N., Salari D., Niaei A., Khataee A. (2006). Photocatalytic degradation of the herbicide erioglaucine in the presence of nanosized titanium dioxide: Comparison and modeling of reaction kinetics. J. Environ. Sci. Health Part B.

[49] Azar F., Yang H.-B., Venault L., Faure S. (2012). Degradation of erioglaucine dye under γ-irradiation. Procedia Chem..

[50] Jain R., Sikarwar S. (2008). Photodestruction and COD removal of toxic dye erioglaucine by TiO_2_-UV process: Influence of operational parameters. Int. J. Phys. Sci..

[51] Shenoy S., Jang E., Park T.J., Gopinath C.S., Sridharan K. (2019). Cadmium sulfide nanostructures: Influence of morphology on the photocatalytic degradation of erioglaucine and hydrogen generation. Appl. Surf. Sci..

[52] Khezrianjoo S., Revanasiddappa H.D. (2015). Electrochemical oxidation of m-cresol purple dye in aqueous media. Water Qual. Res. J. Can..

[53] Chatterjee S., Tyagi A.K., Ayyub P. (2014). Efficient Photocatalytic Degradation of Rhodamine B Dye by Aligned Arrays of Self Assembled Hydrogen Titanate Nanotubes. J. Nanomater..

[54] Sundararajan M., Sailaja V., Kennedy L.J., Vijaya J.J. (2017). Photocatalytic degradation of Rhodamine B under visible light using nanostructured zinc doped cobalt ferrite: Kinetics and mechanism. Ceram. Int..

[55] Zhao G., Zou J., Li C., Yu J., Jiang X., Zheng Y., Hu W., Jiao F. (2018). Enhanced photocatalytic degradation of rhodamine B, methylene blue and 4-nitrophenol under visible light irradiation using TiO_2_/MgZnAl layered double hydroxide. J. Mater. Sci. Mater. Electron..

[56] Xue Y., Wu Z., He X., Yang X., Chen X., Gao Z. (2019). Constructing a Z-scheme Heterojunction of Egg-Like Core@shell CdS@TiO_2_ Photocatalyst via a Facile Reflux Method for Enhanced Photocatalytic Performance. Nanomaterials.

[57] Luna-Flores A., Morales M.A., Agustín-Serrano R., Portillo R., Luna-López J.A., Pérez-Sánchez G.F., Luz A.D.H.-d.l., Tepale N. (2019). Improvement of the Photocatalytic Activity of ZnO/Burkeite Heterostructure Prepared by Combustion Method. Catalysts.

[58] Liu Z., Song Y., Wang Q., Jia Y., Tan X., Du X., Gao S. (2019). Solvothermal fabrication and construction of highly photoelectrocatalytic TiO_2_ NTs/Bi_2_MoO_6_ heterojunction based on titanium mesh. J. Colloid Interface Sci..

[59] Jo H.J., Jung S.H., Kim H.-J. (2004). Synthesis and Hydrogen-Bonded Supramolecular Assembly of Trans-Dihydroxotin (IV) Tetrapyridylporphyrin Complexes. Bull. Korean Chem. Soc..

