# Supplementary material for: Hybrid Composite of Sn(IV)-Porphyrin and Mesoporous Structure for Enhanced Visible Light Photocatalytic Degradation of Organic Dyes
Source: Molecules. 2023 Feb 16;28(4):1886. doi: 10.3390/molecules28041886 (PMC9966349; doi:10.3390/molecules28041886)
Supplement: Supplementary file 1 [file molecules-28-01886-s001.zip › molecules-2182599-supplementary.pdf]

Supplementary Materials

Hybrid Composite of Sn(IV)-Porphyrin and Mesoporous Structure for Enhanced Visible Light Photocatalytic Degradation of Organic Dyes

Nirmal Kumar Shee, Beom-Hyeok Park and Hee-Joon Kim*

Department of Chemistry and Bioscience, Kumoh National Institute of Technology
61 Daehak-ro, Gumi 39177, Republic of Korea

List of contents

Table S1. Zeta potentials and mobilities of SiO_2, **SnP@SiO$_2$**, MCM−41, **SnP@MCM−41**, and **SnP**.

Table S2. Band-gap energies (E_g) calculated by the Tauc plot method.

Table S3. Porosities of MCM−41 and **SnP@MCM−41**.

Figure S1. XRD patterns of **SnP**, **SnP@SiO$_2$**, and **SnP@MCM−41**.

Figure S2. High-resolution FE-SEM images of (a) SiO_2, (b) **SnP@SiO$_2$**, (c) MCM−41, (d) **SnP@MCM−41**, and (e) **SnP**.

Figure S3. EDS mapping of **SnP@SiO$_2$**.

Figure S4. EDS mapping of **SnP@MCM−41**.

Figure S5. Hydrodynamic particle size distributions of **SnP**, SiO_2, MCM−41, **SnP@MCM−41**, and **SnP@SiO$_2$** obtained by the DLS method.

Figure S6. Adsorptivities of **SnP**, **SnP@MCM−41**, and **SnP@SiO$_2$** for the EG dye.

Figure S7. Time-dependent absorption spectra of EG in the presence of **SnP@MCM−41** under visible light irradiation.

Figure S8. Various external effects on the degradation of the EG dye in the presence of **SnP@MCM−41** under visible light irradiation. . ($[Na_2EDTA]_0$ = $[p\text{-BQ}]_0$ = $[^t\text{BuOH}]_0$ = 2 mM, pH = 7.0, T = 298 K).

Figure S9. Kinetics for the photocatalytic degradation of EG under visible light irradiation by the photocatalysts **SnP**, **SnP@MCM−41**, and **SnP@SiO$_2$**.

Figure S10. Time-dependent absorption spectra of the RhB dye in the presence of **SnP@MCM−41** under visible light irradiation.

Figure S11. Kinetics for the photocatalytic degradation of RhB under visible light irradiation by the photocatalysts **SnP**, **SnP@MCM−41**, and **SnP@SiO$_2$**.

Figure S12. Time-dependent absorption spectra of the MCP dye in the presence of **SnP@MCM−41** under visible light irradiation.

Figure S13. Kinetics for the photocatalytic degradation of MCP under visible light irradiation by the photocatalysts **SnP**, **SnP@MCM−41**, and **SnP@SiO$_2$**.

Figure S14. Typical catalytic cycle (up to 10 cycles) for the photocatalyst **SnP@MCM−41** for the degradation of the EG dye.

Figure S15. Comparison of FT-IR spectra of **SnP@MCM−41** before and after the degradation of the EG dye.

Figure S16. Comparison of FE-SEM images of **SnP@MCM−41** before and after the degradation of the EG dye.

Figure S17. Effect of the temperature on the degradation of the EG dye by **SnP@MCM−41**.

Figure S18. Effect of the pH of the solution of the EG dye for photodegradation by **SnP@MCM−41**.

Figure S19. Effect of the concentration of the EG dye for photodegradation by **SnP@MCM−41** (20 mg) within 90 min of visible light irradiation.

Figure S20. Effect of the amount of **SnP** adsorbed onto **SnP@MCM−41** composite for photocatalytic degradation of EG dye, where X = mmol of **SnP** per gram of **MCM−41**.

Figure S21. ESI−MS spectrum (negative ion mode) of the reaction mixture of EG in the presence of **SnP@MCM−41** after 45 min of visible light irradiation.

Table S1. Zeta potentials and mobilities of SiO_2, **SnP@SiO₂**, MCM−41, **SnP@MCM−41**, and **SnP**.

Sample	pH	Zeta potential (mV)	Mobility (cm^2/Vs)
SiO_2	7	-23.80	-1.856×10^{-4}
**SnP@SiO_2**	7	-17.56	-1.370×10^{-4}
MCM−41	7	-30.66	-2.382×10^{-4}
**SnP@MCM−41**	7	-27.53	-2.139×10^{-4}
**SnP**	7	-20.41	-1.558×10^{-4}

Table S2. Band-gap energies (E_g) calculated by the Tauc plot method.

Material	Band-gap energy (eV)
SiO_2	8.51
**SnP@SiO_2**	2.64
MCM−41	5.79
**SnP@MCM−41**	2.53
**SnP**	2.85

Table S3. Porosities of MCM−41 and **SnP@MCM−41**.

Sample	BET surface area (m^2/g)	Pore size (nm)	Pore volume (cm^3/g)
MCM−41	729.546	1.5362	0.551
**SnP@MCM−41**	648.686	1.5319	0.512

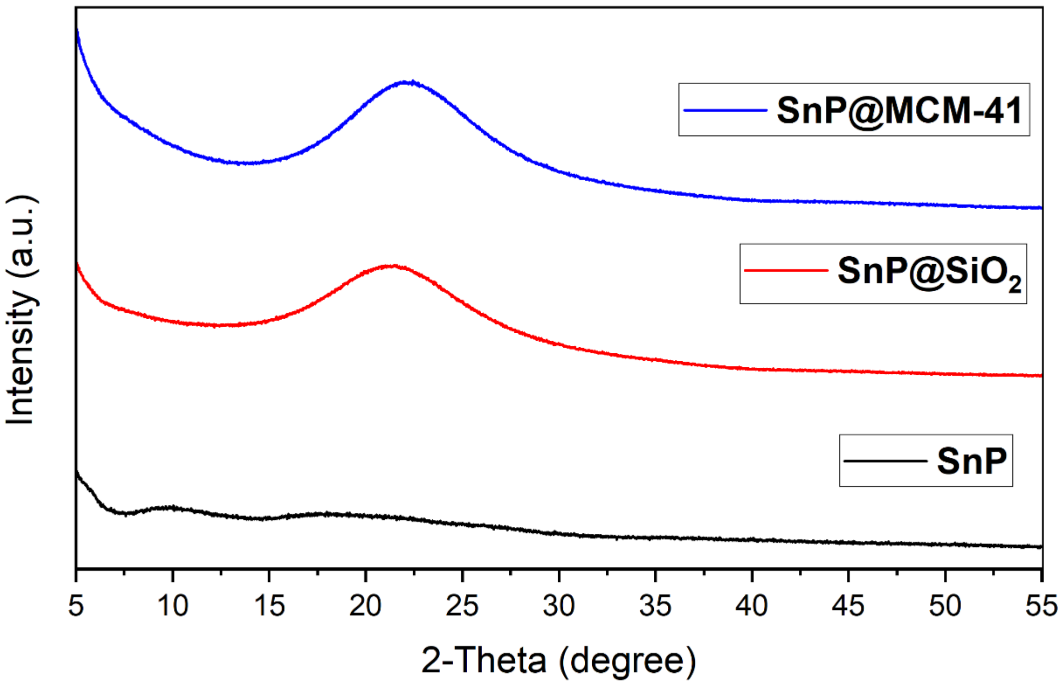

Figure S1. XRD patterns of **SnP**, **SnP@SiO$_2$**, and **SnP@MCM−41**.

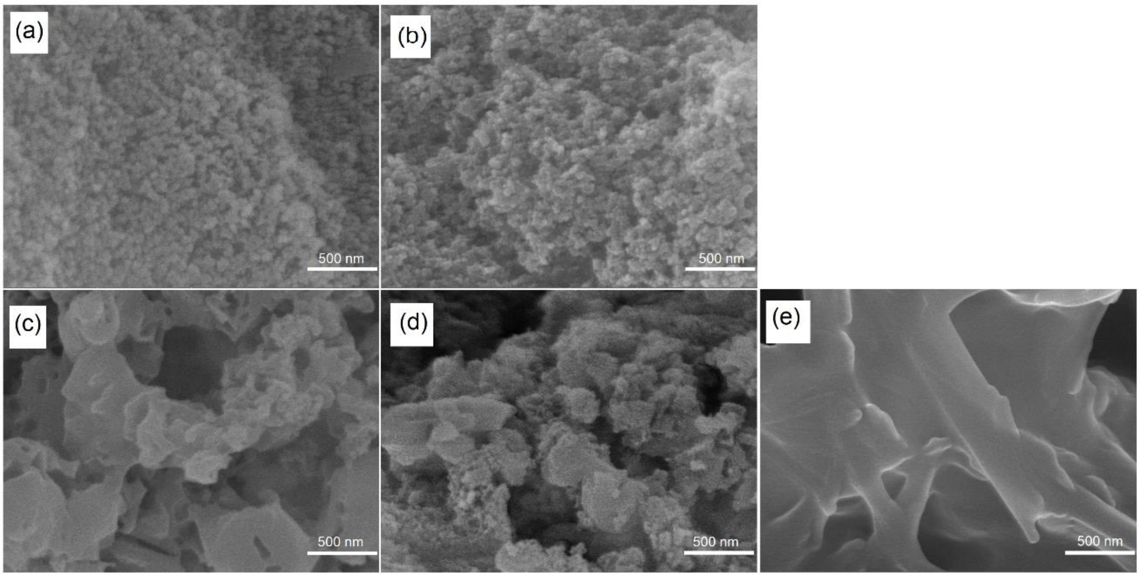

Figure S2. High-resolution FE-SEM images of (a) SiO$_2$, (b) **SnP@SiO$_2$**, (c) MCM−41, (d) **SnP@MCM−41**, and (e) **SnP**.

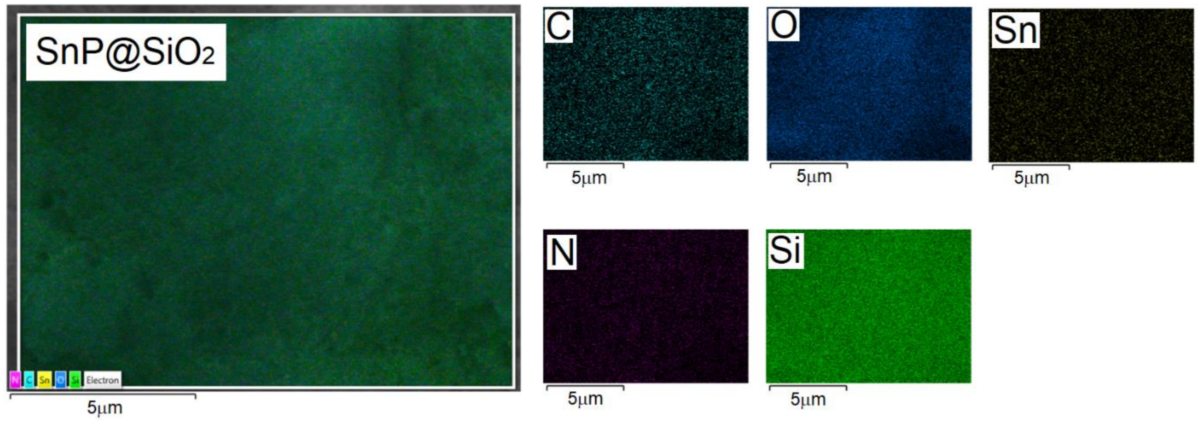

Figure S3. EDS mapping of **SnP@SiO$_2$**.

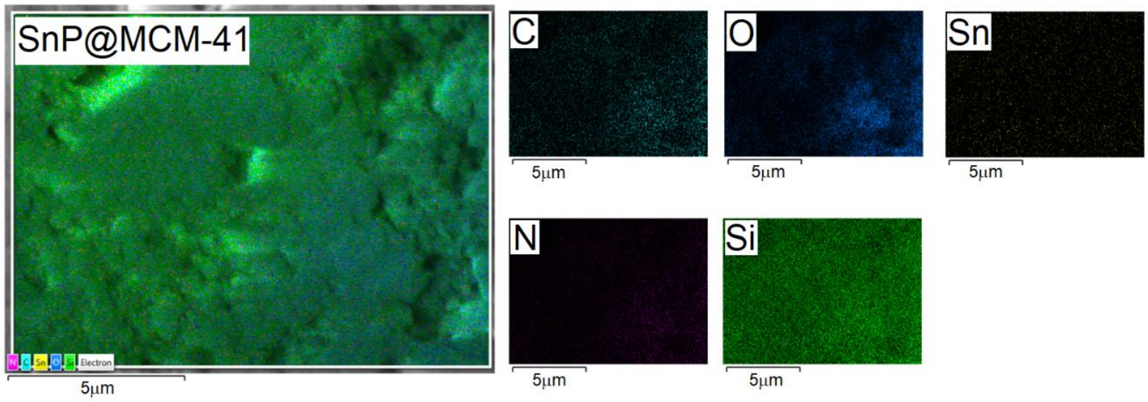

Figure S4. EDS mapping of **SnP@MCM−41**.

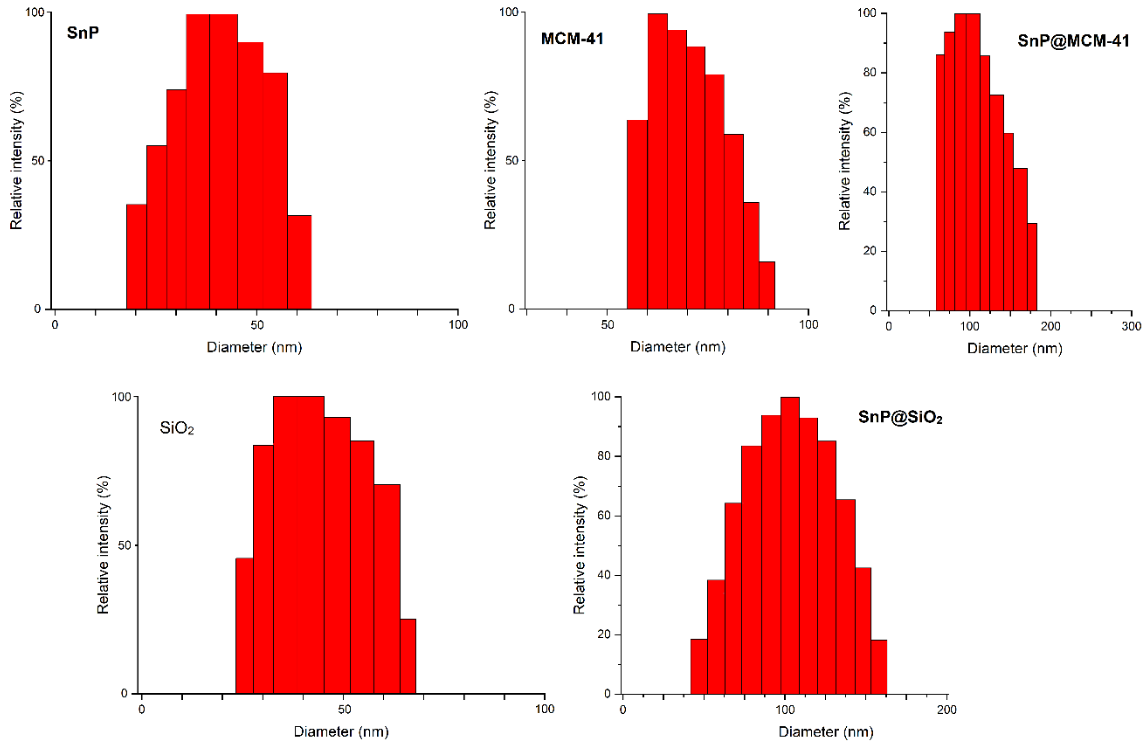

Figure S5. Hydrodynamic particle size distributions of **SnP**, SiO_2, MCM−41, **SnP@MCM−41**, and **SnP@SiO_2** obtained by the DLS method.

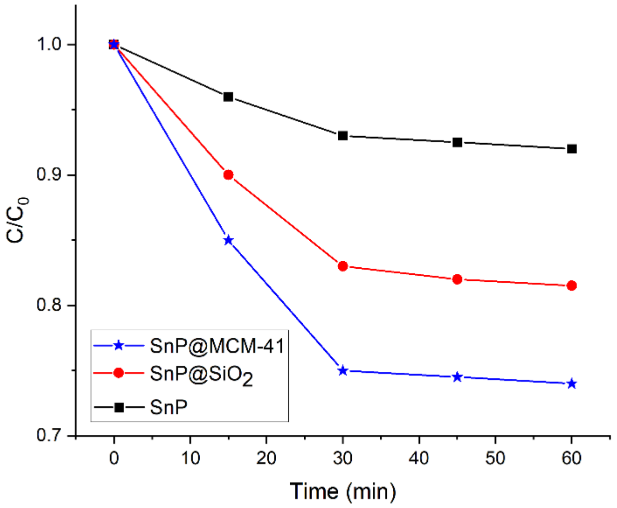

Figure S6. Adsorptivities of **SnP**, **SnP@MCM−41**, and **SnP@SiO_2** for the EG dye.

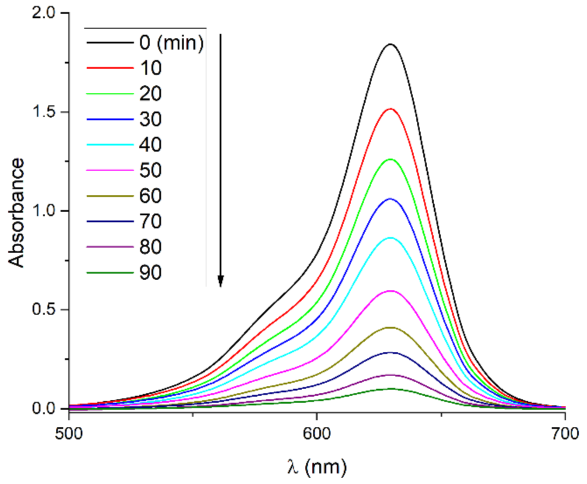

Figure S7. Time-dependent absorption spectra of EG in the presence of **SnP@MCM−41** under visible light irradiation.

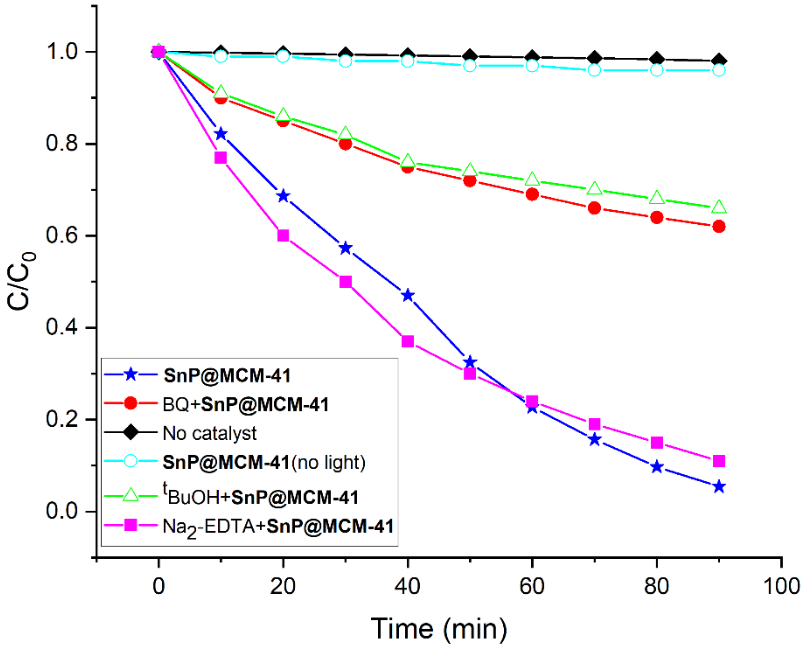

Figure S8. Various external effects on the degradation of the EG dye in the presence of **SnP@MCM−41** under visible light irradiation. ($[Na_2EDTA]_0$ = $[p\text{-BQ}]_0$ = $[^t\text{BuOH}]_0$ = 2 mM, pH = 7.0, T = 298 K).

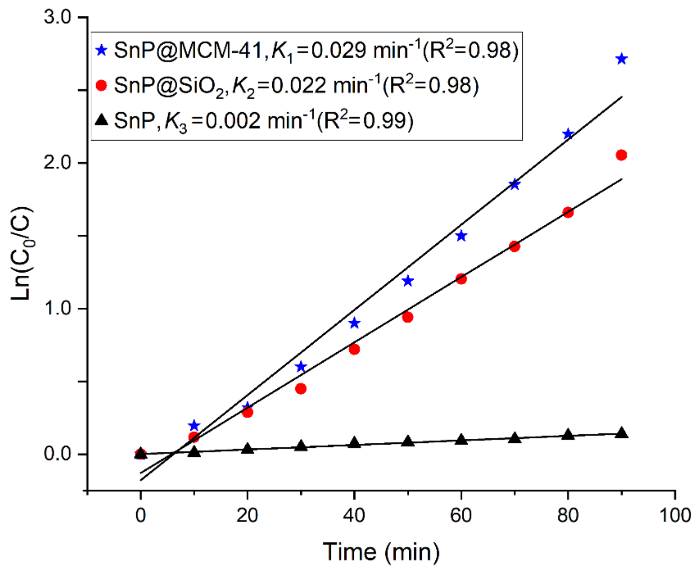

Figure S9. Kinetics for the photocatalytic degradation of EG under visible light irradiation by the photocatalysts **SnP**, **SnP@MCM−41**, and **SnP@SiO2**.

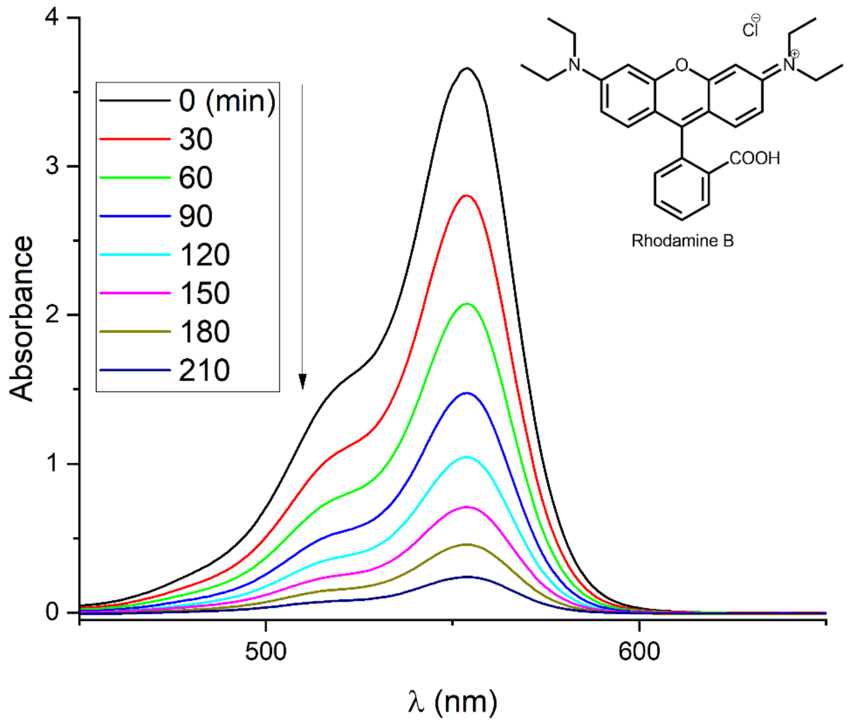

Figure S10. Time-dependent absorption spectra of the RhB dye in the presence of **SnP@MCM−41** under visible light irradiation.

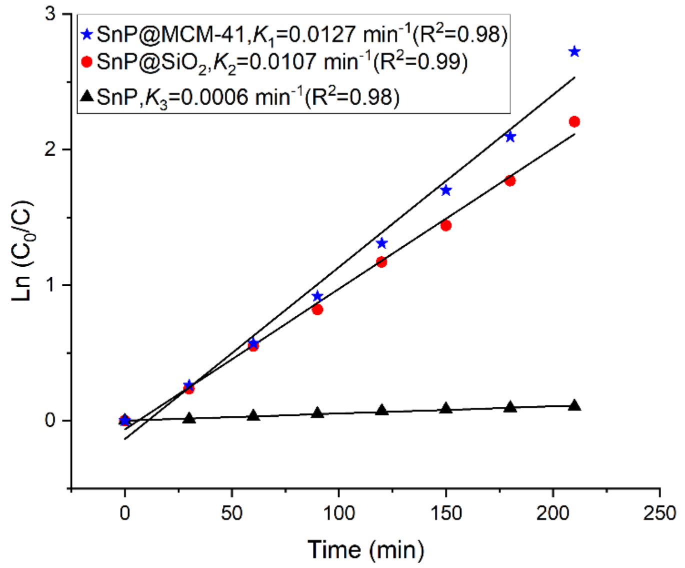

Figure S11. Kinetics for the photocatalytic degradation of RhB under visible light irradiation by the photocatalysts **SnP**, **SnP@MCM−41**, and **SnP@SiO2**.

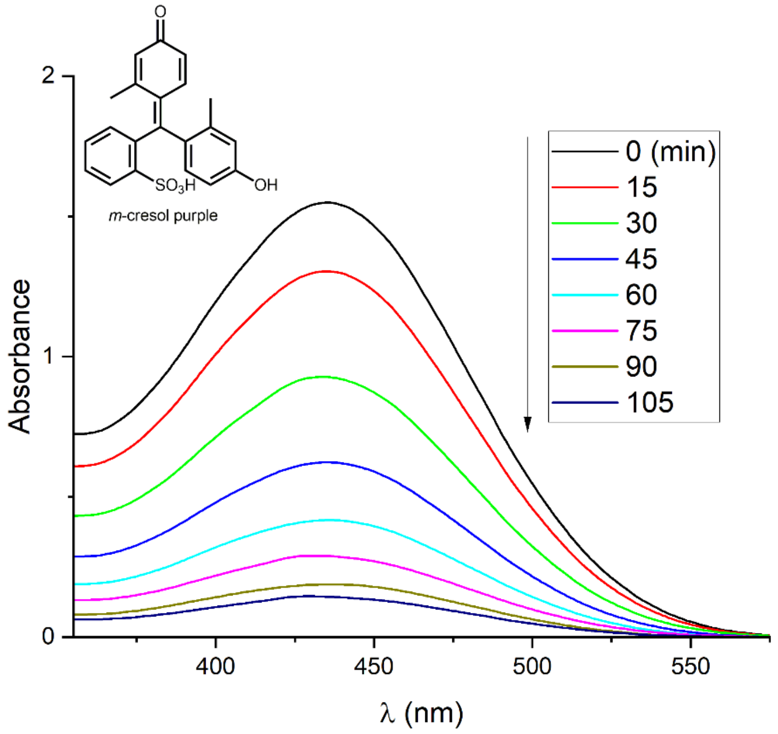

Figure S12. Time-dependent absorption spectra of the MCP dye in the presence of **SnP@MCM−41** under visible light irradiation.

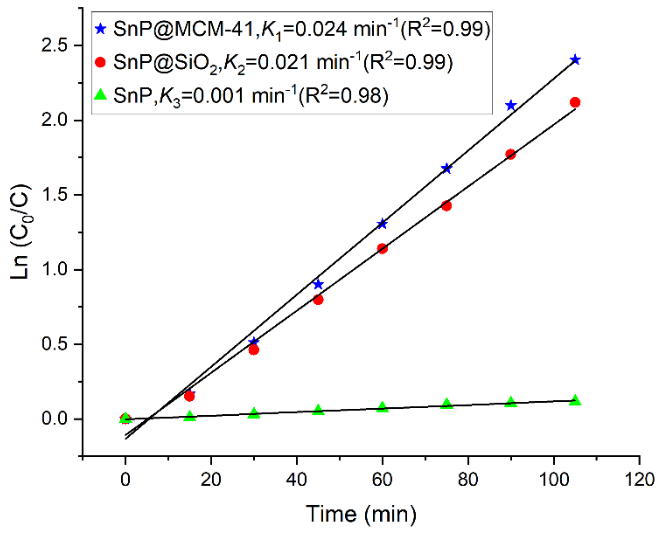

Figure S13. Kinetics for the photocatalytic degradation of MCP under visible light irradiation by the photocatalysts **SnP**, **SnP@MCM−41**, and **$SnP@SiO_2$**.

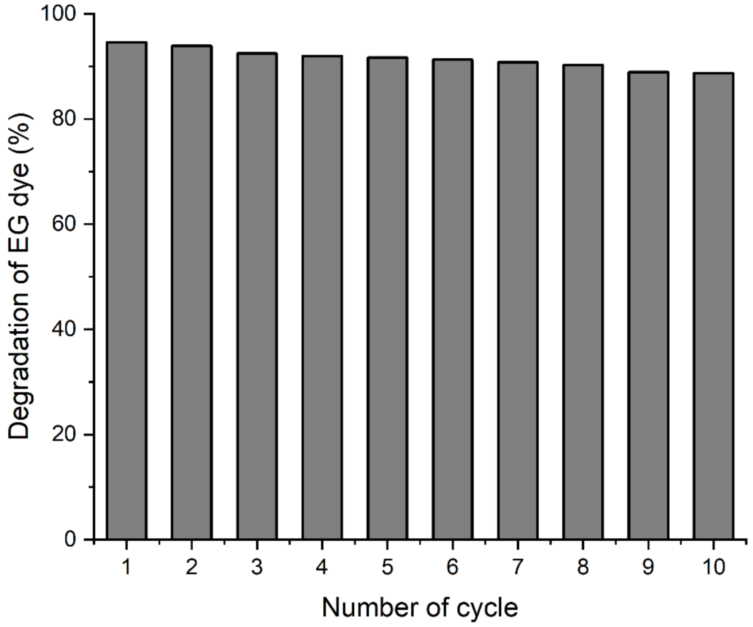

Figure S14. Typical catalytic cycle (up to 10 cycles) for the photocatalyst **SnP@MCM−41** for the degradation of the EG dye.

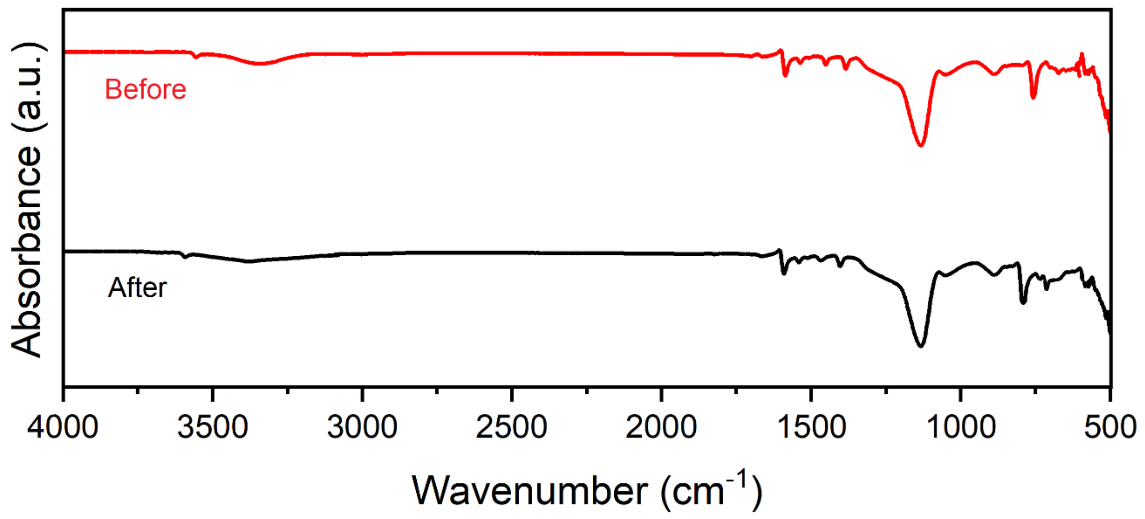

Figure S15. Comparison of FT−IR spectra of **SnP@MCM−41** before and after the degradation of the EG dye.

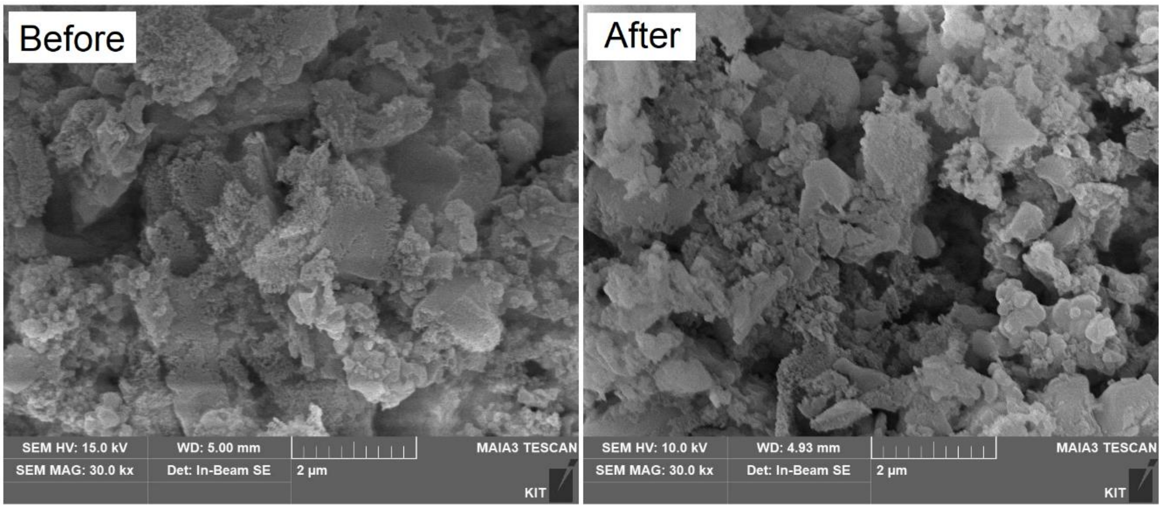

Figure S16. Comparison of FE−SEM images of **SnP@MCM−41** before and after the degradation of the EG dye.

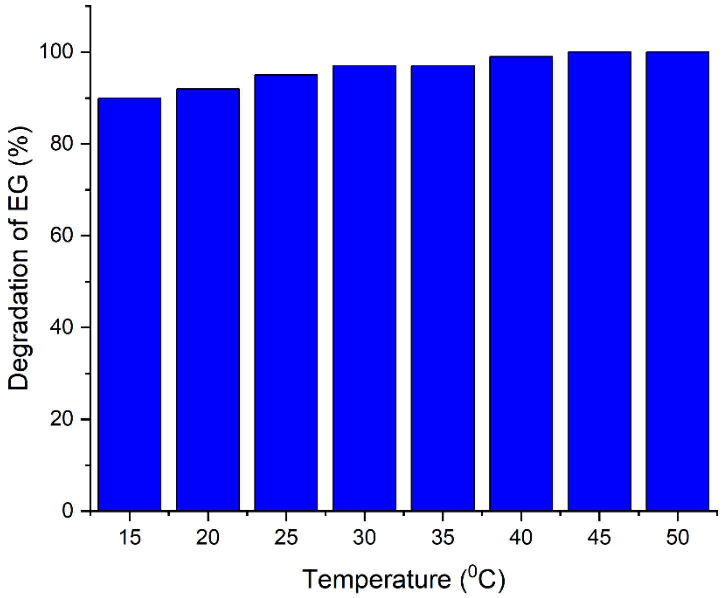

Figure S17. Effect of the temperature on the degradation of the EG dye by **SnP@MCM−41**.

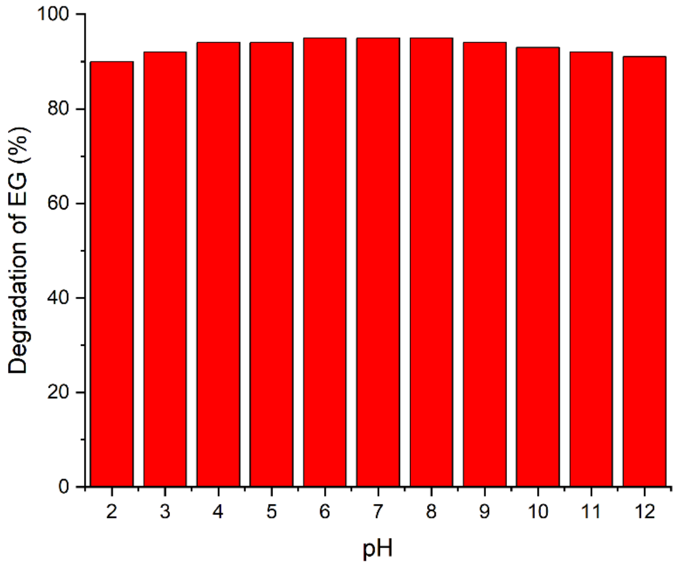

Figure S18. Effect of the pH of the solution of the EG dye for photodegradation by **SnP@MCM−41**.

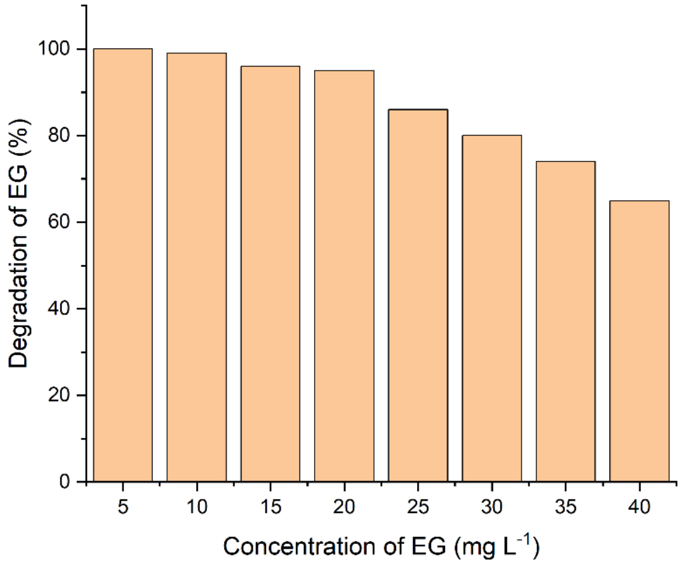

Figure S19. Effect of the concentration of the EG dye for photodegradation by **SnP@MCM−41** (20 mg) within 90 min of visible light irradiation.

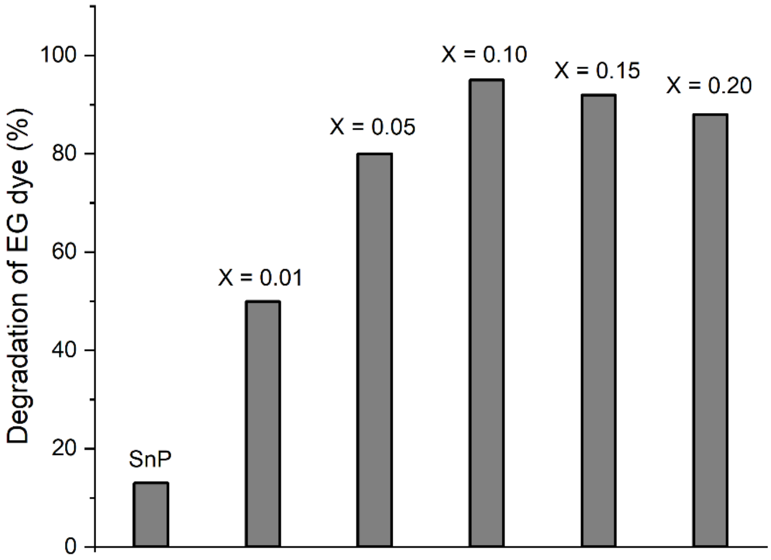

Figure S20. Effect of the amount of **SnP** adsorbed onto **SnP@MCM−41** composite for photocatalytic degradation of EG dye, where X = mmol of **SnP** per gram of MCM−41.

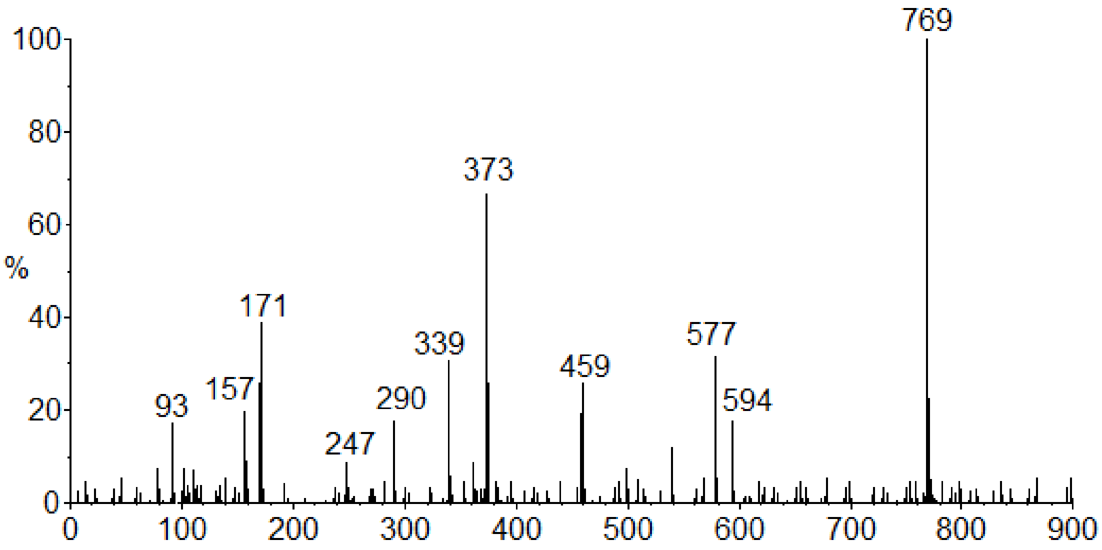

Figure S21. ESI−MS spectrum (negative ion mode) of the reaction mixture of EG in the presence of **SnP@MCM−41** after 45 min of visible light irradiation.
